# Loss of fibronectin fiber tension is inherent to ECM remodeling in human myocarditis and post-inflammatory fibrosis

**DOI:** 10.1016/j.mbplus.2025.100182

**Published:** 2025-10-02

**Authors:** Krishna Chander Sridhar, Julia Mehl, Karin Klingel, Mario Thiele, Sophie Van Linthout, Carsten Tschöpe, Georg N Duda, Viola Vogel

**Affiliations:** a Julius-Wolff-Institute (JWI), Berlin Institute of Health – Charité-Universitätsmedizin Berlin, Berlin, Germany; b Laboratory of Applied Mechanobiology, Department of Health Sciences and Technology, ETH Zürich, Zürich, Switzerland; c Institute of Cardiopathology and Infectious Pathology, Universitätsklinikum Tübingen, Tübingen, Germany; d Berlin Center for Regenerative Therapies (BCRT), Berlin Institute of Health (BIH) – Charité-Universitätsmedizin Berlin, Berlin, Germany; e Deutsches Herzzentrum der Charité, Klinik für Kardiologie, Angiologie und Intensivmedizin, CVK, Augustenburger Platz 1, 13353 Berlin, Germany; f German Center for Cardiovascular Research (DZHK), Partner Site Berlin, Charité-Universitätsmedizin Berlin, Berlin, Germany

**Keywords:** Myocarditis, Extracellular matrix fiber tension, Inflammatory and fibrotic cell niches, Fibronectin-binding peptide FnBPA5, Mechanobiological signature

## Abstract

**Background:**

Viral myocarditis (MC) is associated with extracellular matrix (ECM) remodeling and involves excessive deposition of collagen and other ECM proteins by cardiac fibroblasts, potentially leading to scarring and ECM stiffening, which may subsequently contribute to impaired cardiac functions. Clarifying whether changes in ECM fiber tension are detectable during either the acute or scarring phase could provide a novel, mechanistically relevant mechanobiological signature.

**Objectives:**

Our goal was to ask whether ECM mechano-markers can be identified in the myocardium, beyond excessive collagen fiber deposition, that are associated with the acute infection or the pathological scarring of the human heart, and how this might be associated with the infiltration of macrophages.

**Methods:**

Left-ventricular (LV) endomyocardial biopsies were obtained from patients (N = 39) with acute myocarditis MC (N = 21) including COVID-19 (N = 4) patients suspected with myocarditis MC and/or impaired LV function, dilated cardiomyopathy (N = 6), inflammatory dilated cardiomyopathy (N = 12) to specify diagnosis and treatment options. Endomyocardial biopsies were analyzed for viral genomes, immune cells infiltration and ECM remodeling. Fibronectin fiber tension was assessed using the fibronectin-binding tension-sensor (FnBPA5), while collagen fiber deposits were visualized using second harmonic generation (SHG) microscopy.

**Results:**

While fibronectin fibers were tensed in healthy hearts, histological staining with our novel tension probe FnBPA5 showed that fibronectin fibers had lost their tension in distinct loci in all patient groups. In the acute inflammatory phase (MC), loci with untensed fibronectin fibers were found in close proximity to infiltrated macrophages. In already dilated hearts, biopsies which presented low densities of infiltrated macrophages, thick collagen I/III fiber bundles as visualized by SHG were found in proximity to untensed fibronectin fibers and myofibroblasts, which together are indicative of fibrotic ECM niches where the inflammation has subsided. Comparison of clinical diagnostic and experimental histological data showed clear correlations between altered ECM niche properties and cardiac function deterioration.

**Conclusions:**

Our data suggest that relaxed fibronectin fibers are a recurrent feature of myocarditis and associate with measures of cardiac dysfunction. These findings suggest that fibronectin fiber tension might be a mechanobiological signature that warrants validation in larger, longitudinal cohorts and evaluation of in-vivo measurability.

## Highlights

• Tensional alterations of ECM fibers due to myocarditis were never assessed before
• While fibronectin fibers are stretched in healthy hearts, they were relaxed in proximity to macrophages in acute myocarditis (MC and COVID-19) and to fibrotic lesions in dilated cardiomyopathy (DCM)
• Comparing experimental and clinical data revealed a correlation between the existence of loci enriched in relaxed fibronectin fibers and the deterioration of cardiac functions
• Loss of fibronectin fiber tension is inherent to viral myocarditis and is associated with, or might even drive the fibrotic progression

## Graphical abstract

**Figure:**
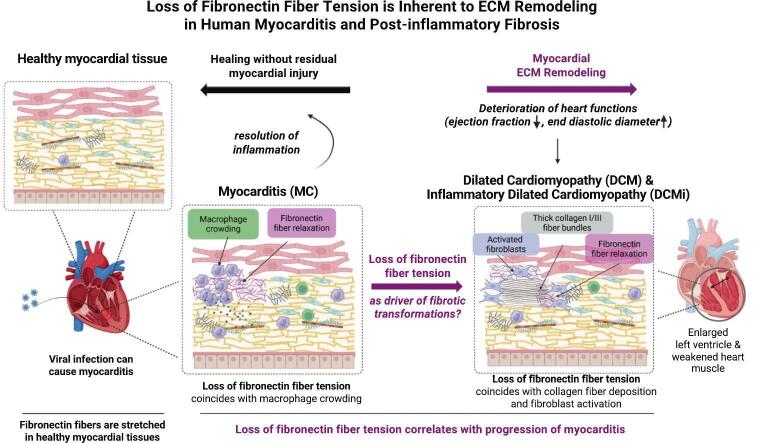
Research Highlight Overview Figure.

## Introduction

Myocarditis (MC) refers to the inflammation of myocardial tissues in the heart triggered by infections from bacteria, fungi, protozoans, viruses as well by toxic substances and drugs [1]. These causative agents can initiate direct cardiac injury or indirectly trigger inflammatory responses through cytokine-mediated cytotoxicity or autoimmune response against host cardiac proteins [1,2]. MC has a complex pathogenesis and is associated with diverse clinical outcomes ranging from healing without myocardial injury and loss of cardiac functions, to severe injury and scarring promoting the progression to heart failure [[3], [4], [5]]. MC pathogenesis is further complicated by the viral persistence, chronic inflammation and ventricular remodeling. Despite the availability of wide range of diagnostic modalities such as analysis of blood markers, histological analysis of patient endomyocardial biopsies, detection of viral genomes, and assessment of cardiac functions that provide valuable details of the pathology, non-invasive imaging techniques are well suited to detect more severe cases and include chest X-ray, Echocardiography or MRI, whereby MRI is considered the gold standard for diagnosing MC, due to its sensitivity to detect edema, fibrosis, and inflammation in the myocardium. In the initial hours to days of myocarditis, however, patients may have elevated biomarkers in the blood due to the onset of myocyte damage with only minor or subclinical changes on imaging or ECG, thereby providing clinicians with a critical window of opportunity for disease detection and to initiate interventions before the onset of irreversible ventricular remodeling and systolic dysfunction. Upon the onset of the inflammatory phase, ECM remodeling is driven by the imbalanced actions of matrix metalloproteinases (MMPs), counteracted by tissue inhibitors of metalloproteinases (TIMPs), and other proteolytic systems [6]. Beyond the well described scarring-associated ECM stiffening, the understanding of how extracellular matrix (ECM) remodeling affects disease progression and why some patients with MC heal without residual injury, while others suffer from dilated cardiomyopathy (DCM) or chronic inflammatory cardiomyopathy (DCMi) is still unclear [[1], [2], [3], [4],[7], [8], [9]].

In the healthy heart muscle, cardiomyocytes (CMs) and cardiac fibroblasts co-exist, whereby they are responsible for heart development and maintenance, as well as for secreting growth factors, cytokines and other signaling molecules [10]. The majority of cardiac cells in the adult mammalian heart are non-myocytes [11]. Cardiac fibroblasts are the primary cells responsible for the production, maintenance and remodeling of the extracellular matrix (ECM) in the heart, an essential structural feature compensated by enhanced tissue repair activity of activated fibroblasts, which locally transform into highly contractile myofibroblasts thereby safeguarding the structural integrity of the myocardial tissue by establishing collagenous scarring [[12], [13], [14]]. As the heart is one of the least self-regenerating organs in the body, successful tissue repair is a critical determinant of maintaining essential heart function after injury [15]. Myofibroblastic phenotypes often continue to be active post healing, with a positive-feedback loop resulting in continuous synthesis and assembly of collagen I and III fibers, ultimately leading to cardiac fibrosis [14,16,17]. Progressive cardiomyocyte loss and the onset of fibrosis accompany DCM, a heterogeneous group of diseases (excluding coronary artery disease) clinically characterized by left ventricular dilation and tissue remodeling. The progression to DCM is often attributed to persistence of virus and/or chronic inflammatory processes in the myocardium [9,18] referred to as DCMi [7]. Currently, diagnosis of acute MC, DCM and DCMi are based on analyses of patient endomyocardial biopsies that reveal the inflammatory status [1]. Endomyocardial biopsies reveal immune cell compartments, inflammasomes (e.g. NLRP3), miRNAs as well as viral genome, cardiac muscle damage and fibrosis [1,19,20]. While the focus was largely on immune cell compartments and CM-fibroblast electro-mechanical crosstalk, the importance of the ECM in regulating cardiovascular functions is finding increasing recognition in recent years [21,22].

The myocardial ECM is not only a dynamically remodeled meshwork of structural proteins to anchor cells, but it also serves a reservoir to harbor growth factors, cytokines, chemokines, proteases, protease inhibitors and microRNAs [23]. The ECM is thus a crucial organizer of cellular microenvironments in which cardiomyocytes, fibroblasts, leukocytes and cardiac vascular cells reside and communicate with each other, physically and biochemically [[24], [25], [26]]. This complex crosstalk of cardiac cells and the ECM preserves cardiac functions during homeostasis; however, it is also responsible for pathological ECM remodeling following myocardial injury [27,28]. In the myocardial ECM, apart from collagen I/III fibers, which have been the major focus in previous studies of cardiac scarring, other structural (eg. fibronectin, elastin) and non-structural matricellular proteins (eg. tenascin, osteopontin, SPARC) play pivotal roles in regulating myocardial tissue homeostasis and remodeling [7,24,26,27,[29], [30], [31], [32], [33], [34]]. Recent work has underscored the relevance of ECM remodeling in virus-induced cardiac inflammation, including COVID-19-associated myocarditis. Here, a shift from macrophage-driven inflammation to fibrosis was shown to involve profound ECM reorganization, highlighting the importance of structural ECM dynamics in disease progression [35]. Fibronectin fibrillogenesis is required for collagen-1 and thrombospondin-1 deposition and maintenance of fibronectin fibrils requires ongoing remodeling processes [36]. This process is mechano-tuned as the onset of collagen fibrillogenesis itself is dependent on fibronectin fiber tension [37]. While no probes were available until recently to quantify fibronectin fiber strain in healthy versus diseased organs, we recently introduced a bacterial-derived peptide FnBPA5, whose multivalent binding epitope to the N-terminal Fibronectin type 1 domains (FnI_2-5_) is destroyed by fiber stretching [[38], [39], [40]]. After thoroughly validating this tension probe *in vitro* [41] and in healthy organs versus tumor tissues, a critical role of fibronectin fiber tension in maintaining tissue integrity has emerged [42,43]. While fibronectin fibers were highly stretched in healthy mice organs, including the heart. In contrast, a major fraction of fibronectin fibers had lost their tension in tumor tissues, virus-infected lymph nodes and late inflammatory stages of *Salmonella*-infected mouse intestines [42,44,45]. These data suggest that maintenance of fibronectin fiber tension is crucial for tissue homeostasis and signifies, if lost, pathological ECM remodeling that is paralleled by the infiltration of immune cells [[44], [45], [46]]. Therefore, we asked here whether fibronectin fiber tension is altered in MC and how this might relate to MC progression as alterations in ECM fiber tension can potentially regulate the healing processes.

To study how the tensional signature of fibronectin fibers might get altered in myocarditis, in the acute inflammatory phase as well as in loci where scars are left behind, and how this correlates with clinical parameters, we analyzed endomyocardial biopsies from 39 patients. We performed histological analyses of the left ventricular endomyocardial biopsies from patients representing different stages of MC, namely acute MC (including COVID-19), as well as DCM, and DCMi patients (Suppl. Table 1). The presence and spatial distribution of immune cell populations and fibrillar ECM components were analyzed in detail. For the first time, we analyzed the tensional states of fibronectin fibers in human heart tissues using our well validated Fibronectin-binding peptide FnBPA5 as fiber tension sensor [[39], [40], [41], [42]]. To identify fibrotic regions, second harmonic generation (SHG) microscopy was performed to visualize the presence of thick collagen fiber bundles, a hallmark of fibrotic pathologies [47]. Based on these analyses, we describe here the presence of distinct ECM signatures in myocardial tissues at different stages of the disease progression, with respect to ECM fiber tension and composition. We finally discuss the potential clinical significance of these altered ECM niche signatures, including inflammatory and fibrotic ECM niches that might be associated with tuning the transition of the inflammation phase towards a healing versus a fibrotic outcome.

## Results

### Immunohistochemical analyses revealed the presence of CD68^+^ macrophages and CD3^+^ T cells in endomyocardial biopsies of myocarditis patients

To investigate ECM transformations during the progression of inflammation in viral myocarditis (MC), we first analyzed the clinical diagnostic data of three patient groups at Charité, namely acute MC, dilated cardiomyopathy (DCM) and inflammatory dilated cardiomyopathy (DCMi) that represent different stages of disease progression to understand the difference between the groups. Additionally, we also analyzed COVID-19 patients suspected with MC due to cardiac infection of SARS-COV2 virus or SARS-COV2 cytokine-induced cardiac inflammatory response. The patient biopsy collection, histological preparation and diagnostic test procedures and data are provided in the Method Section 5.1 and 5.7.

**Fig. 1:**
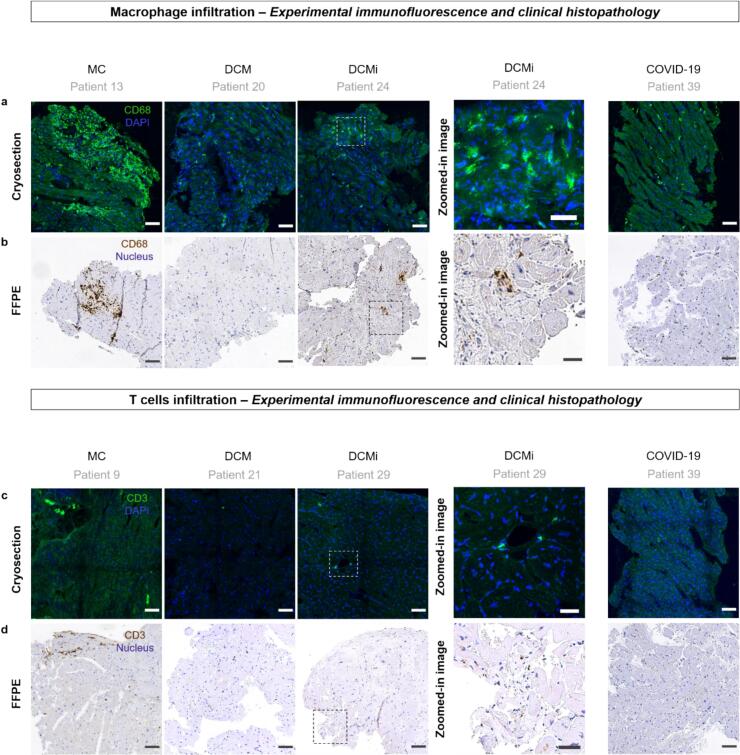
Immuno-histochemical and clinical histopathological evaluation of left ventricular biopsies across patient groups: a) IHC images show CD68^+^ macrophages (green) in biopsies from the left ventricle of patients from acute myocarditis (MC), dilated cardiomyopathy (DCM), inflammatory dilated cardiomyopathy (DCMi) and COVID-19 patient groups, N = 39; MC-17, DCM-6, DCMi-12, COVID-19–4; refer Suppl. Fig. S1 for further images of macrophage infiltration in myocardial tissues across patient groups. b) Enzymatic immunostained images of formalin-fixed paraffin embedded (FFPE) sections from the corresponding patient biopsies, but from different cross-sections, showing CD68^+^ macrophages (brown), N = 30; MC-13, DCM-5, DCMi-11, COVID-19–1. c) IHC images showing CD3^+^ T cells (green) in biopsies from patients across the patient groups, N = 37; MC-16, DCM-5, DCMi-12, COVID-19–4; refer Suppl. Fig. S2 for further examples of T cells infiltration in myocardial tissues across patient groups d) Enzymatic immunostained images of FFPE sections from the corresponding patient biopsies, but from different cross-sections, showing CD3^+^ T cells (brown) in tissues across the patient groups, N = 30; MC-13, DCM-5, DCMi-11, COVID-19–1. Scale bars: Tile images (left): 100 µm, enlarged images (right): 50 µm. (For interpretation of the references to colour in this figure legend, the reader is referred to the web version of this article.)

Clinically, MC is histo-pathologically diagnosed based on immune cell infiltrates – especially macrophages and T cells (presence of > 14 leucocytes/mm^2^ of the biopsy tissue area including up to 4 monocytes/mm^2^ with the presence of CD3 positive T-lymphocytes > 7 cells/mm^2^) [1]. Therefore, we firstly performed immunohistochemistry (IHC) on tissue cryosections to study the presence as well the spatial distribution of macrophages and T cells using the pan-macrophage surface marker CD68 and pan-T cell surface marker CD3, respectively [48]. IHC analyses showed the presence of macrophages across the patient groups (N = 39; MC-17, DCM-6, DCMi-12, COVID-19–4) (Fig. 1a). More examples of macrophage infiltrates in tissues across the different patient groups along with corresponding zoom-in images are shown in Suppl. Fig. S1. We then compared our IHC stainings with the clinical histopathology images (used for diagnosis) of patient endomyocardial biopsies (formalin-fixed paraffin embedded, FFPE section) from the same patients but from adjacent cross-sections. Comparison with histopathology images from the patient diagnosis confirmed the presence of macrophage infiltrates in the tissues for all patient groups for N = 30, with images shown for patients MC-13, DCM-5, DCMi-11, COVID-19–1 (Fig. 1b). Similarly, comparison with histopathology images from the patient diagnosis confirmed the infiltration of T cells in the tissue from all patient groups, with images shown for patients N = 37; MC-16, DCM-5, DCMi-12, COVID-19–4 (Fig. 1c) and N = 30; MC-13, DCM-5, DCMi-11, Covid-19–1 (Fig. 1d). Given the high heterogeneity within each tissue sample, as expected, large tissue sections are shown in the main figures complemented by selected zoom-in images at higher resolution for regions of interest in Suppl. Fig. S2.

### Clinical histopathology and cardiac function data showed clear differences between patient groups and their blood parameters revealed high heterogeneity

**Fig. 2:**
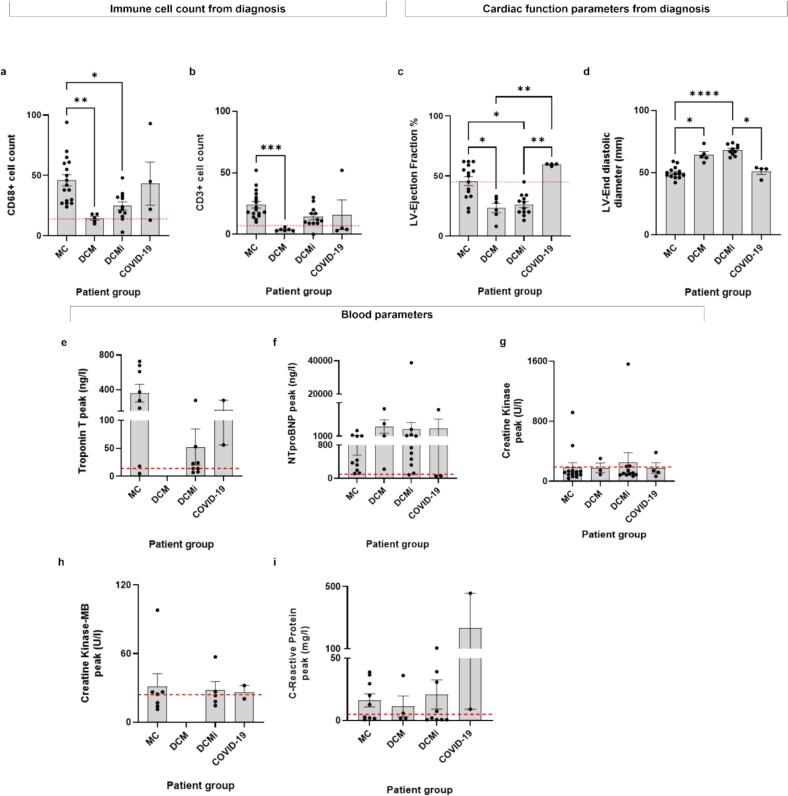
Clinical histopathology and cardiac function analyses. a) Plot depicting CD68^+^ macrophage counts across the patient groups. b) Plot depicting CD3^+^ T cell counts across the patient groups. Both CD68 + and CD3 + cells were counted manually based on immunohistochemistry stainings as shown in Fig. 1. The cells were normalized to cells per sq. mm as tissue sizes are variable. c) Plot depicting the ejection fractions (EF, % of blood volume ejected from the ventricle for each contraction) across the patient groups. d) Plot depicting the left ventricular end diastolic diameters (LVEDD, in mm, measure of left ventricular enlargement) across the different patient groups. e) Plot depicting blood Troponin T (TnT) peak levels in patients across the groups. f) Plot depicting blood N-terminal prohormone of brain natriuretic peptide (NTproBNP) peak levels in patients across the groups. g) Plot depicting creatine kinase (CK) peak levels across the patient groups. h) Plot depicting creatine kinase-myocardial band (CK-MB) peak levels across the patient groups. i) Plot depicting high sensitivity C-reactive protein peak (hsCRP) levels across the patient groups. The threshold level for diagnosis is marked by a red dotted line. (Statistical analysis in (a-i): Kruskal-Wallis test of one-way ANOVA; Statistical significance level set to α = 0.05, * P ≤ 0.05, ** for P ≤ 0.01, *** for P ≤ 0.001, **** for P ≤ 0.0001). (For interpretation of the references to colour in this figure legend, the reader is referred to the web version of this article.)

Following IHC analysis for immune cell infiltration, we quantitatively assessed and compared the immune cell counts and corresponding cardiac function parameters across the patient groups. The macrophage and T cell counts obtained from the clinical histopathological analysis of all endomyocardial biopsies (Fig. 2, Fig. 2) revealed that the macrophage count (mean ± s.e.m.) was significantly higher in MC patients (46 ± 5 cells/mm^2^) as compared to DCM (15 ± 2 cells/mm^2^) and the DCMi (25 ± 3 cells/mm^2^) groups. Further, the macrophage counts were higher in the MC and DCMi groups, which represent tissues with ongoing inflammation, compared to the DCM group. The macrophage counts in COVID-19 patients (43 ± 18 cells/mm^2^) were comparable to the MC group but with high heterogeneity. Similarly, the T cell counts were significantly higher in MC (24 ± 3/mm^2^) patients compared to the DCM (4 ± 0 cells/ mm^2^) patients. In DCMi and COVID-19 patients, the T cell counts were 14 ± 2 cells/mm^2^ and 16 ± 24 cells/mm^2^, respectively. Similar to the macrophage counts, the T cell counts were highly heterogeneous in the COVID-19 group. Moreover, the T cell counts of both MC and DCMi groups were higher than in the DCM group.

The cardiac function parameters were also analyzed for all the patient groups based on the clinical data from echocardiography [1]. The ejection fraction (EF) is a measure of the total blood volume ejected from the heart for each contraction. The normal EF in healthy humans ranges from 52-72 % for males, and 54–74 % for females [49]. The EF (mean ± s.e.m.) was significantly higher in the early phase of myocarditis (MC, 46 ± 4 %) compared to advanced phases (DCM, 23 ± 4 % and DCMi, 26 ± 3 %) indicating deteriorating cardiac output with disease progression (Fig. 2c). In COVID-19 patients, the EF was 60 ± 1 % indicating a well-preserved cardiac function. The left ventricular end diastolic diameter (LVEDD) measures the left ventricular enlargement. The normal LVEDD in healthy humans is ≤ 56 mm for men, and ≤ 51 mm for women, while LVEDD in dilated hearts is higher than 56 mm for men and 51 mm for women [50]. The LVEDD (mean ± s.e.m.) was significantly higher for the DCM (64 ± 3 %) and DCMi (68 ± 1 %) groups compared to the acute MC (50 ± 1 %) and COVID-19 group (51 ± 2 mm) indicating enlargement/dilatation of ventricles with disease progression (Fig. 2d). With a normal range of LVEF and LVEDD and an increased immune cell count, COVID-19 patients represented acute MC.

Although endomyocardial biopsies are a gold standard for the diagnosis of myocardial inflammation and are used to identify the presence of viruses, the time of infection can typically not be traced back once patients come to the clinic and present heart symptoms. To understand the status of inflammatory and myocardial damage, blood parameters troponin T (TnT), creatine kinase (CK), creatine kinase myocardial band (CK-MB), high sensitivity C-reactive protein (hsCRP) and N-terminal pro–B-type natriuretic peptide (NT-proBNP) peaks were analyzed for all the patient groups [51] (Fig. 2e-i). Unlike the immune cell count and cardiac function parameter analyses, the blood marker analyses did not reveal a significant difference between the patient groups. Some markers such as TnT, NTproBNP and hsCRP, showed a high degree of intra-group heterogeneity. For example, the levels of c-reactive protein were significantly different in the patient groups ranged broadly in acute MC (0.7 – 38.9 mg/l; N = 9), DCM (1.7 – 36.2 mg/l; N = 4), DCMi (0.6 – 106.1 mg/l; N = 9) and COVID-19 (9.1 – 457.8 mg/l; N = 2). Given that the C-reactive protein is produced by the liver in response to inflammation with its systemic levels rising during acute inflammation and falling when inflammation is resolved [52], the wide range of values argues that patients within the same group differ in the phase and/or severity of inflammation. Taken together, the analysis of patient diagnostic data shows that patients when clinically grouped based on histopathology and cardiac function tests (MC, DCM, DCMi and COVID-19) can display complex sub-phenotypes as suggested by the analyses of blood markers.

### Inflamed myocardial tissues across patient groups showed major ECM remodeling with major loss of fibronectin fiber tension

**Fig. 3:**
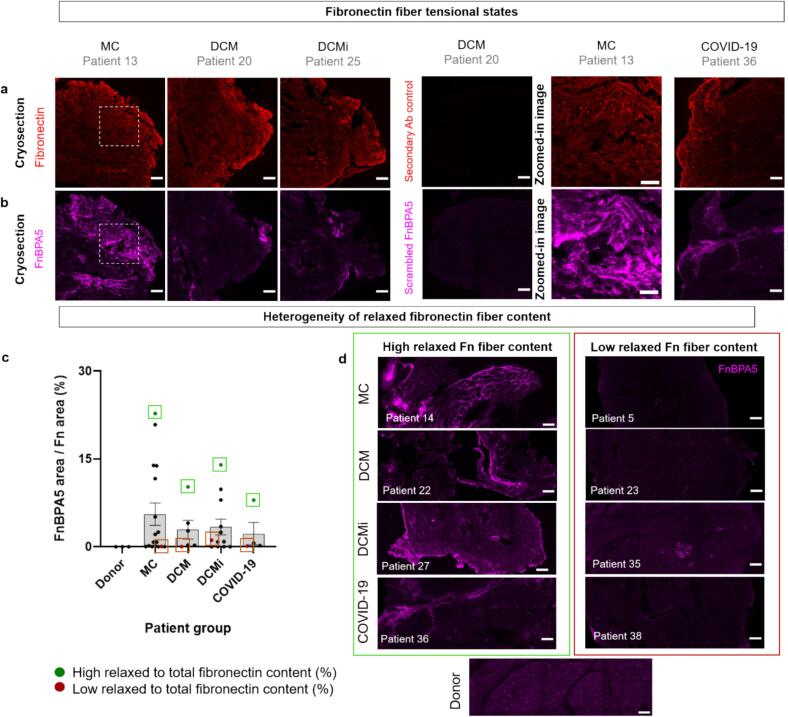
Loci in which fibronectin fibers had lost their tension in inflamed myocardial tissues. a) Immunofluorescence images of fibronectin (polyclonal antibody, red) within myocardial tissues present throughout the tissue cross-section area. b) Corresponding stains from the same tissue sections showing loci of FnBPA5 peptide signal (magenta) indicating the locations of untensed fibronectin fibers; Scale bars: Tile images: 200 µm, enlarged images: 50 µm.; refer Suppl. Fig. S3 and S4 for more images showing relaxed fibronectin fibers in inflamed myocardial tissues across patient groups and corresponding zoom-in images c) Plot depicting the ratio of pixels positive in untensed fibronectin fibers versus total fibronectin fiber pixels in the whole tissue for samples from the different patient groups and healthy donors. d) The histological stainings revealed varying levels of untensed fibronectin fiber pixel content within each patient group: (left) images of tissues with the highest content of untensed to total fibronectin content (%) (shown within green boxes in Fig. 3c) and (right) images of tissues with lowest content of untensed to total fibronectin content (%) (shown within red boxes in Fig. 3c). Scale bars: 100 µm. Patient samples: N total = 39, N: MC = 17, DCM = 6, DCMi = 12, COVID-19 = 4, Donor samples = 3. Statistical analysis in (c) Kruskal-Wallis test of one-way ANOVA; Statistical significance level set to α = 0.05, * P ≤ 0.05, ** for P ≤ 0.01, *** for P ≤ 0.001, **** for P ≤ 0.0001. (For interpretation of the references to colour in this figure legend, the reader is referred to the web version of this article.)

While inflammation is associated with local remodeling of the cardiac ECM [26,29,53,54], nothing was known how inflammation affects the tension of cardiac ECM fibrils. We thus also analyzed the tensional states of fibronectin fibers across patient groups. We used a polyclonal fibronectin antibody that stains all fibronectin fibers, together with the fibronectin-binding peptide tension sensor (FnBPA5) [41] which only binds to fibronectin fibers that have lost their tension. Histological analyses in the myocardial tissues across patient groups showed distinct loci rich in untensed fibronectin fibers, marked by the binding of Cy5-FnBPA5 peptide (Fig. 3, Fig. 3). More examples of fibronectin fiber relaxation in inflamed myocardial tissues across patient groups along with corresponding zoom-in images are shown in Suppl. Fig. S3. As fibronectin fibers in healthy heart tissues are highly stretched, the presence of untensed fibronectin fibers indicates loci of abnormal ECM [42]. A scrambled-Cy5-FnBPA5 peptide (having the same amino acid residues as Cy5-FnBPA5 but randomly rearranged) was used as a negative control which does not bind to stretched or untensed fibronectin fibers [41]. The stainings revealed no signal from the scrambled-FnBPA5 peptide as opposed to a strong signal from the FnBPA5 peptide indicating specific binding of the peptide to fibronectin fibers. Further, in some tissues, untensed fibronectin fibers were found along the tissue edges. To exclude the possibility of artifacts at tissue edges due to flipping or sectioning altered fibronectin fiber tension was analyzed in the intact tissue areas (Suppl. Fig. S4). Out of 39 tissues analyzed, loci of untensed fibronectin fibers were found in 29 tissues (MC-12/17, DCM-5/6, DCMi-8/12 and COVID-19–4/4). Notably, most samples that were clinically tested positive for viruses in the molecular diagnosis (PCR for viral genome in biopsy; refer Suppl. Table 1 for patient clinical diagnostic data) were concomitantly found to contain untensed fibronectin fibers (N = 5/5, MC-4, DCM-0, DCMi-1, COVID-19–0).

To compare the untensed fibronectin fiber content across patient groups, a quantitative analysis was performed as described in Method Section 5.5.2. Due to the variability in tissue areas of the samples, the untensed fibronectin fiber content was plotted as a ratio of total tissue fibronectin pixels of the whole tissues. The untensed fibronectin fiber pixel content was relatively higher in tissues from the MC (6 ± 2 % of total tissue area) and DCMi (3 ± 1 %) groups which represent acute and chronic inflammation, respectively, when compared to the DCM (3 ± 1 %) and COVID-19 (2 ± 2 %) groups (Fig. 3c). In tissues from healthy donors (N = 3; treated with immunosuppressant prior to transplant), the untensed fibronectin fiber content was negligible. Further, histological analysis of the untensed fibronectin fiber content showed a high variability of untensed fibronectin fiber content within each of the patient groups, covering a large range for acute MC (0–23 %), DCM (0–10 %), DCMi (0–14 %) and COVID-19 (0–8 %) (Fig. 3d). To represent the heterogeneity within patient groups, the images of tissues with highest untensed to total fibronectin fiber content (%) for each patient group (shown within green boxes in Fig. 3c) are shown in Fig. 3d (left), while the images of tissues with lowest untensed to total fibronectin fiber content (%) in each group (shown within red boxes in Fig. 3c) are shown in Fig. 3d (right). Given the high heterogeneity, likely because viral infections act locally, a statistical significance in untensed to total fibronectin fiber content (%) was not found between the patient groups. Nevertheless, the histological analysis of the patient tissues with the FnBPA5 probe revealed local areas of major ECM remodeling characterized by loss of fibronectin fiber tension across all patient groups.

### Inflamed myocardial tissues across patient groups showed enhanced fibrillar collagen deposition as probed by SHG

**Fig. 4:**
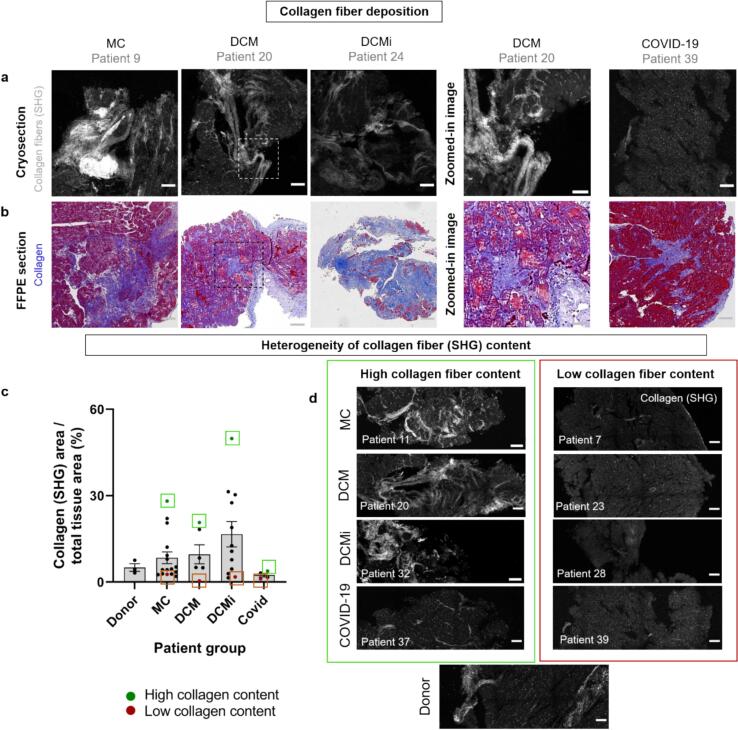
Fibrillar Collagen deposits (SHG-positive) in inflamed myocardial tissues. a) Second harmonic generation (SHG) microscopy images show thick collagen fiber deposits (grey) in myocardial tissues across the patient groups. b) Clinical histopathological Masson’s trichrome stainings of cardiac muscle fibers in red and collagen fibers in blue from all patient groups. The FFPE tissues are from the same patients but display different cross-sections. Scale bars: Tile images: 200 µm, enlarged images: 50 µm. c) Plot depicting the pixel ratios of fibrillar collagen deposits (SHG) versus the total tissue areas for samples from the patient groups and healthy donors (N = 39). Patient tissues with the highest collagen fiber content versus the lowest collagen fiber content are shown within green and red boxes respectively for individual groups. d) SHG microscopy images revealed varying levels of collagen fiber content within each patient group: (left) images of tissues with the highest content of collagen fibers in each patient group (within green boxes in Fig. 4c) and (right) images of tissues with low/no collagen fiber content (within red boxes in Fig. 4c). It is unclear whether some of these fibrotic lesions were preexisting to the onset of myocarditis. Scale bars: 100 µm. Patient samples: N total = 39, N: MC = 17, DCM = 6, DCMi = 12, COVID-19 = 4, Donor samples = 3); Statistical analysis in (c): Kruskal-Wallis test of one-way ANOVA; Statistical significance level set to α = 0.05, * P ≤ 0.05, ** for P ≤ 0.01, *** for P ≤ 0.001, **** for P ≤ 0.0001. (For interpretation of the references to colour in this figure legend, the reader is referred to the web version of this article.)

As unresolved inflammation often results in progressive fibrosis and scar tissue formation [12,13], second harmonic generation (SHG) microscopy was performed to detect the presence of collagen I/III fibers and hence the onset of fibrosis. SHG microscopy analysis revealed the presence of collagen fiber deposits in tissues across patient groups (Fig. 4a). Further images of tissues with fibrous collagen deposits (SHG) are shown in Suppl. Fig. S5. In some tissues, the fibrosis was focal, while in others it was perivascular or interstitial [55] in nature (Suppl. Table 1). To further validate our observations, we compared the SHG images with histopathology images of FFPE tissues from the same patients but in different tissue cross-section with Masson’s trichrome stainings. Similar to our observation from SHG, collagen fibers were found in the tissues across all the patient groups (Fig. 4b). Comparing the histological stains and SHG images revealed the local presence of both untensed fibronectin fibers and collagen fiber bundles in several tissues. Particularly in the DCM group all samples that contained untensed fibronectin fibers also contained SHG-positive collagen fiber bundles (N = 5/5) indicating a correlation between fibronectin fiber relaxation and fibrosis in the advanced stage of disease.

To compare the collagen fiber content in different patient groups, a quantitative analysis was performed (Methods section 5.5.2). The SHG-positive pixels normalized to the total tissue areas was slightly higher in the DCM (10 ± 3 % of total tissue area) and DCMi (17 ± 4 %) groups that represent advanced stages of the disease compared to the MC group (8 ± 2 %) and COVID-19 group (2 ± 1 %) (Fig. 4c). Similar to our observations with the untensed fibronectin fiber content, the collagen fiber content was also highly heterogeneous within each patient group ranging between 2–28 % in MC, 0–21 % in DCM, 1–50 % in DCMi and 1–4 % in COVID-19. To represent the heterogeneity within patient groups, the images of tissues with the highest collagen fiber content for each patient group (shown in green boxes in Fig. 4c) are shown in Fig. 4d (left), while the images of tissues with the lowest collagen fiber content in each group (shown in red boxes in Fig. 4c) are shown in Fig. 4d (right). In healthy donor tissues, the collagen fiber content was low and comparable to that of MC. A statistical significance in the collagen fiber content was not found between the patient groups, probably for the same reason that viral infections act locally, but a trend is seen.

The histological analysis of the ECM using the FnBPA5 probe and SHG imaging revealed locations of major ECM remodeling, albeit to a different extent and with different spatial patterns. Further, the loss of fibronectin fiber tension coincided with the deposition of thick collagen I/III bundles in the inflamed myocardial tissues from MC, DCM, DCMi and COVID-19 patients which warrants a further investigation of the link between these ECM proteins and their respective roles in the progression of MC. In line with the observation from the clinical blood marker analyses, the heterogeneity in ECM remodeling patterns per patient group argues for a different phase and/or severity of inflammation in patients that have received the same clinical diagnosis. The ECM remodeling patterns and their heterogeneities within each patient group could indicate distinct patterns of immune cell infiltration, and concomitant differences in ECM degradation and remodeling. This could have a long-term effect on the healing versus scarring outcome in patients.

### Loss of fibronectin fiber tension was found in proximity to macrophages during acute inflammation, as well as next to fibrotic lesions in the dilated heart

**Fig. 5:**
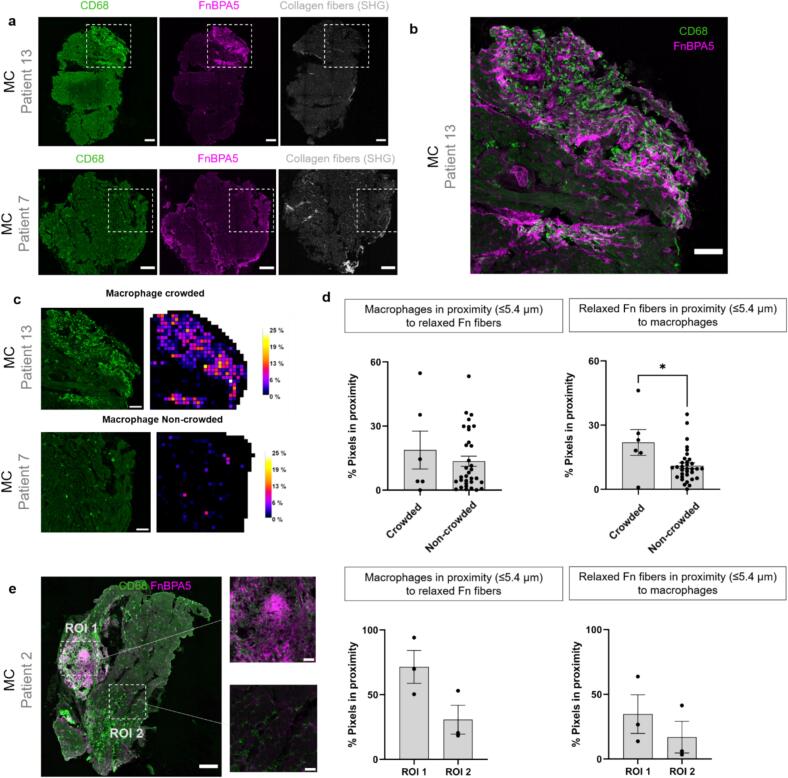
Spatial proximity analysis of macrophages and untensed fibronectin fibers. a) Histological stainings and SHG images from two acute myocarditis (MC) patient tissues show macrophage crowded areas (Patient #13) and those without macrophage crowding (Patient #7). Both tissues contained areas rich in untensed fibronectin (Fn) fibers while the deposition of SHG-positive collagen fiber deposits was not pronounced. b) Enlarged ROI shown in images a of macrophage-crowded loci, providing an overlay of macrophages (green) and untensed fibronectin fibers (magenta). c) Enlarged ROI shown in a for patients #7 and #13 together with the corresponding heatmaps quantifying macrophage density (refer Methods section 5.5.1 for methods set the threshold value for defining tissues that contain macrophage-crowded loci versus macrophage non-crowded tissues). d) Plots depict the percentage of pixels within a distance ≤ 5.4 µm relative to each other: (left) fraction of macrophage pixels near untensed fibronectin fiber pixels, and (right) fraction of untensed fibronectin fiber pixels near macrophage pixels (N = 5 for macrophage crowded tissues and N = 30 for macrophage non-crowded tissues, Mann-Whitney non-parametric test, α = 0.05, *p ≤ 0.05). e) Histological staining shows areas of high (ROI 1) and low (ROI 2) macrophage-density coexisting in the same tissue of an acute myocarditis patient. The plots depict the percentage of pixels positive for untensed fibronectin fibers and macrophages at a distance ≤ 5.4 µm to each other (N = 3, Statistical analysis in (c-d): Mann-Whitney non-parametric test, Statistical significance level set to α = 0.05, * P ≤ 0.05, ** for P ≤ 0.01, *** for P ≤ 0.001, **** for P ≤ 0.0001), Scale bars: 200 µm. (For interpretation of the references to colour in this figure legend, the reader is referred to the web version of this article.)

Histological analyses revealed numerous loci of untensed fibronectin fibers in inflamed myocardial tissues (N = 29, MC-12, DCM-5, DCMi-8, COVID-19–4) (Fig. 3). However, due to high heterogeneity, the content of untensed fibronectin fibers or collagen fibers could not alone or together distinguish between the different stages of disease progression. Therefore, we next investigated how the local spatial pattern of immune cell infiltration correlated with ECM remodeling. In some myocardial tissues, co-staining for macrophages (CD68) and untensed fibronectin fibers (FnBPA5) revealed macrophage-dense areas that were concomitantly enriched in untensed fibronectin fibers, specifically in the MC, DCMi and COVID-19 patient groups (Fig. 5, Fig. 5). If the local fraction of CD68 positive pixels exceeded 0.2 % (mean % of CD68 + pixels per square grid area; refer Methods Section 5.5.1), we defined such regions as macrophage-crowded (Fig. 5c). Across all patients, we quantified the fraction of CD68 and FnBPA5 positive pixels that were ≤ 5.4 μm apart from each other to ask whether there was a preferential localization of macrophages in close proximity to untensed fibronectin fibers (Fig. 5d, left), or vice versa (Fig. 5d, right). In Patient#7 and Patient#13 diagnosed with acute myocarditis, macrophage infiltration and fibronectin fiber relaxation were not associated with SHG-positive collagen fiber deposits, suggesting that the patient’s hearts had not (yet) undergone scarring. In these cases, a positive correlation between untensed fibronectin fiber and macrophage pixels at distances ≤ 5.4 μm relative to each other is found, specifically in tissues that displayed a high macrophage density (N = 6, MC-3, DCMi-2, COVID-19–1). The proximity analyses indeed indicate that inflammation (acute or chronic) characterized by macrophage crowding correlates with locations where fibronectin fibers had lost their tension. In tissues infiltrated by macrophages, the correlation (mean ± s.e.m) of the fraction of CD68 pixels proximal to FnBPA5 pixels is 20 ± 11 %, or vice versa, and 22 ± 7 % respectively. As expected, the correlation is significantly weaker in the non-macrophage crowded tissue areas, with 14 ± 3 % and 10 ± 1 %, for CD68 pixels proximal to FnBPA5 pixels and vice versa. Spatial proximity analysis was also performed on areas or regions of interest (ROIs) within the same tissue as shown in Fig. 5e, which indicated the same trend as in Fig. 5d. This poses the important question whether relaxed fibronectin fibers promote macrophage invasion, directly or indirectly, or whether macrophage crowding contributes to fibronectin fibers losing their tension.

Fibronectin fiber relaxation can result from multiple mechanisms – necrotic cell death which prevents the cells to pull on ECM fibers, proteolytic activity of proteases that cleave certain fibers, or fibrillar collagen deposition that take over major load-bearing functions [39]. Therefore, histological analysis was performed to investigate the presence of proteases that could cleave fibronectin and other ECM fibers to which fibronectin is anchored, specifically matrix metalloproteases. MMP-2 and MMP-9 are both type IV collagenases in addition to other functions. While MMP-9, but not MMP-2, can be seen next to untensed fibronectin fibers in a tissue stain from the MC group (Suppl. Fig. S6a), note that the overall proteolytic activity is regulated by the relative concentrations of proteolytic enzymes counterbalanced by protease inhibitors [45]. It is well established, for example, that the tissue inhibitor of metalloproteinase-1 (TIMP-1) which binds to MMPs is consistently upregulated in myocardial fibrosis and is used as a marker of fibrosis. By inhibiting MMPs, even though they are upregulated during myocardial inflammation, TIMPs play crucial roles in co-regulating tissue remodeling [56].

**Fig. 6:**
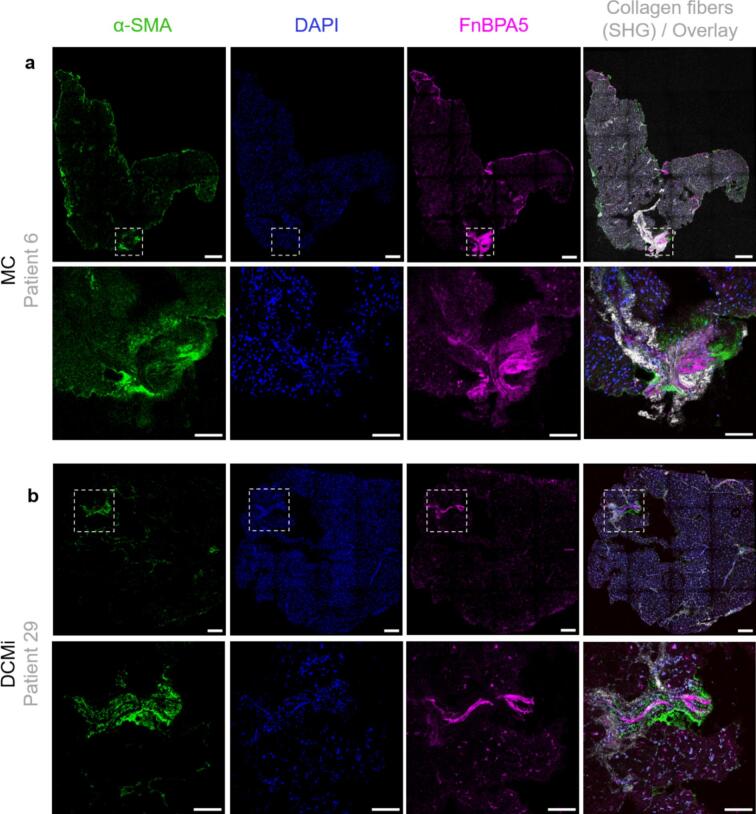
Myofibroblasts, untensed fibronectin fibers and fibrillar collagen deposits (SHG) are localized in close proximity to each other in inflamed myocardial tissues from MC and DCMi patient groups. (a) In a tissue from an acute myocarditis (MC) patient, immunostaining together with SHG imaging show the presence of αSMA^+^ myofibroblasts (green) in areas with untensed fibronectin fibers (magenta) and fibrillar collagen deposits (grey (above). Scale bars: 200 µm. Corresponding enlarged images of the region-of-interest (below). Scale bars: 50 µm. (b) In a tissue from a chronic inflammatory dilated cardiomyopathy patient, immunostaining together with SHG imaging show the presence of αSMA^+^ myofibroblasts in loci with untensed fibronectin fibers and fibrillar collagen deposits (above). Scale bars: 200 µm. corresponding enlarged images of the region-of-interest (below). Scale bars: 50 µm. Refer to Suppl. Fig. S8 for a high-resolution image with myofibroblasts in proximity to areas rich in collagen fibers (SHG) and relaxed fibronectin fibers. (For interpretation of the references to colour in this figure legend, the reader is referred to the web version of this article.)

In some of our patient biopsies (N = 12; MC-4, DCM-4, DCMi-4), fibronectin fiber relaxation was found in proximity to areas of fibrillar collagen deposits in several tissues across patient groups, but not in all. As untensed fibronectin fibers serve as multivalent templates to initiate collagen fiber assembly [37], we next analyzed if untensed fibronectin fibers form niches that are potentially primed for subsequent scarring. To explore fibronectin relaxation beyond the active inflammation, we examined myocardial regions with low macrophage density (<0.2 % CD68 + pixels per square grid area; refer to Methods section 5.5.1) for macrophage infiltration analysis). For tissues in Fig. 6a, the macrophage density was 0.05 % while in Fig. 6b the macrophage-density was 0.12 %, which could either indicate a borderline or an already subsided inflammation. In these tissues that do not show macrophage crowding, the SHG-positive fibrotic lesions are enriched in α-SMA^+^ myofibroblasts, untensed fibronectin fibers and collagen fiber bundles (Fig. 6, Fig. 6 (N = 2)). High resolution images showing myofibroblasts in loci enriched in untensed fibronectin fibers and fibrillar collagen deposits are shown in Suppl. Fig. S8. The presence of SHG-positive fibrotic lesions, while lacking macrophage crowding (macrophage density < 0.2 %), suggests that the inflammation had already subsided in these two patients (Patient #6 and Patient #29), or preexisted due to earlier heart injuries. While these features are not surprising individually, their co-localization in regions devoid of inflammatory infiltration suggests that fibronectin fiber relaxation may persist into the fibrotic phase. We also distinguished across the clinical groupings between patients where the biopsies contained loci of macrophage infiltration (crowded) or not (non-crowded) as summarized in Suppl. Table 5. Indeed, the immune cell (CD68^+^ and CD3^+^) blood counts were much higher in patients where the myocardial tissues showed loci of macrophage infiltration (N = 5), compared to the rest of the tissues (N = 30). The same trend is seen for the levels of inflammatory marker C-reactive protein (CRP) and the muscle damage marker creatine kinase (CK), while the troponin (TropT) levels were much higher in patients where the biopsies did not (yet) show macrophage infiltration (N = 5 for tissues containing macrophage-crowded loci and N = 30 for non-crowded tissues).

These pilot studies are hypothesis generating, with the main goal of determining whether changes in the tensional signature of ECM fibers might have clinical relevance. Our data will hopefully motivate future longitudinal studies that systematically assess changes in fibronectin fiber tension and the infiltration of immune cells using larger group specific cohorts. This approach could help clarify whether the loss of fibronectin fiber tension precedes, coincides with, or follows the resolution of acute inflammation, thereby providing stronger evidence for its role as a marker in pathological tissue remodeling. Despite the high intra-group heterogeneity and the small number of patient samples, our data show that fibronectin fibers have lost their tension in many loci rich in infiltrated macrophages already during the active inflammation phase (MC) (Suppl. Fig. S7b). Moreover, fibronectin fibers lost their tension, at least in some inflamed loci, prior to the onset of excessive collagen fiber deposition which is a major hallmark of scarring. One possible explanation could be that fibronectin fiber relaxation primes cell niches for subsequent fibrotic progression, as untensed fibronectin fibers can serve as templates to induce collagen fibrillogenesis [37].

### Loss of fibronectin fiber tension correlated with increased blood immune cell counts and the deterioration of cardiac function

**Fig. 7:**
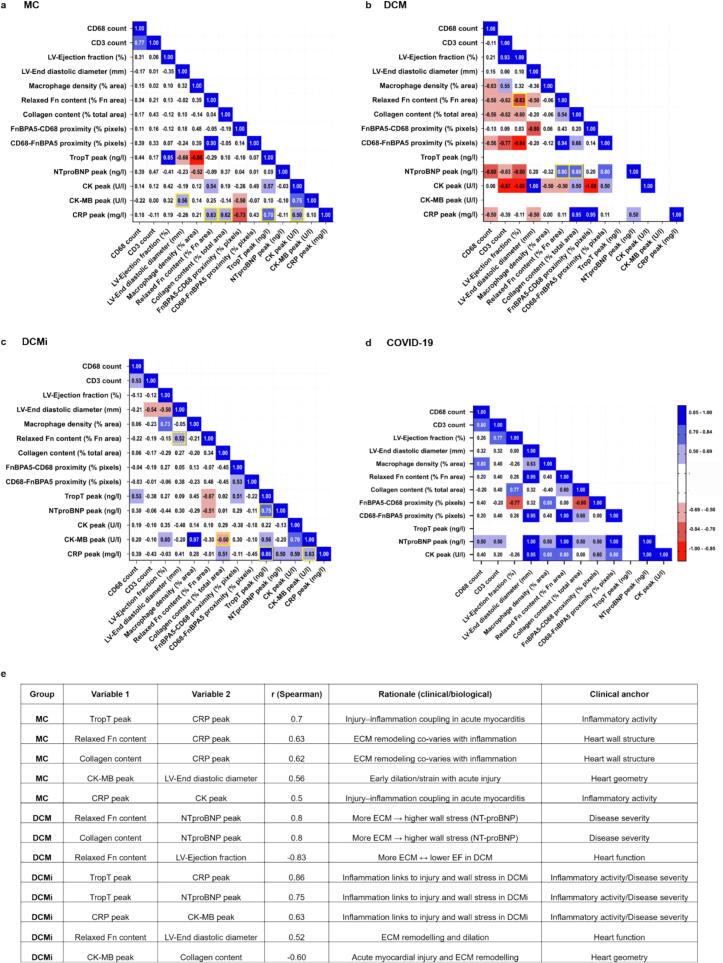
Ranked correlation matrices of clinical diagnostic and experimental data for individual patient groups. (a-d) Spearman rank correlation matrices showing relationships between multiple variables from clinical diagnostic data and immunohistochemical analysis data from the acute MC, DCM and DCMi and COVID-19 groups. The correlation values described in the text are shown as yellow boxes. The correlation coefficients (r) range between −1 and + 1 and the positive/negative signs follow harmonized variable polarity. Only correlations ≥ +0.5 and ≤ -0.5 are shown in red and blue respectively. The most clinically relevant correlations are highlight with a yellow square and are explained below in the table (e) Table with correlation coefficient (r) values of the most relevant correlations between clinical and experimental parameters across all the patient groups (N: MC ≥ 13, DCM-5, DCMi ≥ 10, COVID-19 = 4; Statistical analysis: Spearman rank correlation; Statistical significance level set to α = 0.05, p values for all patient groups are provided in Suppl. Table 3). (For interpretation of the references to colour in this figure legend, the reader is referred to the web version of this article.)

As inflammation, followed in some patients by scarring, is often associated with cardiac remodeling and deterioration of cardiac function, we next analyzed how the immune cell infiltration and ECM remodeling patterns observed in the histological analysis of myocardial biopsies compared to cardiac function and inflammatory markers in the patient’s blood. To gain insights into patient group specific (MC, DCM, DCMi and COVID-19) signatures, we correlated the clinical diagnostic data (blood markers, biopsy data and cardiac function) with microscopic features in myocardial biopsy data regarding macrophage infiltration (% CD68 + pixels to total image pixels), fibronectin fiber relaxation (% FnBPA5 to total fibronectin pixels) and collagen fiber deposition (% of SHG to total image pixels) and finally, the proximity (≤ 5.4 μm) of macrophage to untensed fibronectin fiber pixels, or vice versa (% pixels). The correlation coefficients (r) between the myocardial biopsy, blood and heart function parameters are given here in patient group specific correlation matrices (Fig. 7). All correlation analyses used Spearman’s r. We report effect sizes (r) and two-sided p values (α = 0.05). To aid interpretation, we considered |r|≈0.1–0.3 weak, ≈ 0.3–0.5 moderate, and ≥ 0.5 strong. In the text, we only use the term positive ‘correlation’ when (i) |r| ≥ 0.5 and (ii) if the association is observed for a majority of conceptually linked features within a patient group; otherwise, we refer to them as ‘trends’. Given cohort size and multiple comparisons, analyses are exploratory; p values are listed in Suppl. Table 3. As the blood biomarkers (TropT, CK, CK-MB, CRP) alone or in combination with cardiac function data are used to group the patients (MC, DCM, DCMi), they correlate as expected. Elevation of these earliest blood biomarkers associated with damage of the heart muscle cells typically precedes any detectable changes in cardiac function, including ejection fraction (EF) and left ventricular end diastolic diameter (LVEDD).

The correlation of blood biomarkers and cardiac function data with the untensed fibronectin fiber content was, as hypothesized, different in the MC, DCM and DCMi groups. We also found at the microscopic level that the content of untensed fibronectin fibers correlated with the loci of infiltrated immune cells, as well as with the collagen fiber content as probed by SHG, was significantly different across the patient groups. The untensed fibronectin fiber content positively correlated with the blood levels of the inflammatory marker CRP of the acute MC patients (r = 0.63), in contrast to the DCM (r = 0.11) and DCMi groups (r = -0.01). However, it must be noted that systemic CRP levels indicate inflammation throughout the body and not only in the myocardium. Injury–inflammation pairs (troponin with CRP and CK with CRP; +0.70 and + 0.50 respectively) and ECM–inflammation pairs (untensed fibronectin and collagen fiber content with CRP; +0.63 and + 0.62 respectively) were concordant. A representative link to alterations of the heart geometry was CK-MB peak vs LVEDD (r = +0.56), consistent with early dilation. These correlations together with the histological findings suggest that the acute myocardial inflammation phase coincides with the punctuated losses of fibronectin fiber tension in the cardiac tissues. Troponin can be elevated despite preserved EF in focal myopericarditis or when EF is assessed after the biomarker peak, which explains the positive cross-sectional association (troponin peak vs EF, r = +0.85). We therefore label this item timing-sensitive and exploratory.

As scarring in the post-inflammatory phase can go along with major collagen fiber deposition, mostly collagen I and III, it is important to note that untensed fibronectin fibers are often found next to fibrillar collagen deposits across patient samples. This correlation was stronger in the DCM (r=+0.54) and COVID-19 (r=+0.60) groups compared to MC (r=+0.04) and DCMi (r=+0.34) as expected. Thus fibronectin fiber relaxation seems to be also correlated with fibrotic progression, even though not all locations of relaxed fibronectin fibers necessarily showed fibrillar collagen deposits. It would be intriguing to explore in future studies why this might be the case. In DCM, patients with dilated hearts, a correlation is seen between deteriorating cardiac performance and loci of fibronectin fiber relaxation as well as with fibrillar collagen deposition. Both, the untensed fibronectin fiber content as well as the collagen fiber (SHG) content correlated negatively with the deteriorating left ventricle ejection fraction (LV-EF) (r = -0.83 and r = -0.60 respectively) and strong positive correlation to the heart failure marker NTproBNP (r=+0.80 and r=+0.80 respectively). ECM load tracked with heart wall stress and function (relaxed fibronectin/collagen vs NT-proBNP, positive; vs EF, negative), was in line with chronic ECM remodeling. These correlations indicate that fibronectin fiber relaxation goes along with fibrotic progression and deterioration of cardiac ejection function.

In the DCMi group, a remarkable correlation exists between the presence of macrophage-crowded loci (chronic inflammation) and cardiac muscle damage. The macrophage pixel density in microscopic loci of macrophage crowding is correlated with cardiac muscle damage markers as detected in the patient’s blood, i.e. the CK-MB level (r=+0.97) in DCMi. The correlation between the inflammatory marker CRP and CK-MB was also high (r=+0.63). Further, the correlation between untensed fibronectin fiber content and LVEDD was stronger in the DCMi (r = 0.52) group compared to the MC and DCM groups. This indicates that a chronic inflammation in DCMi is associated with ECM remodeling, cardiac damage and cardiac function deterioration. However, the sequence of macrophage crowding and loss of fibronectin fiber tension in such a chronic setting has to be carefully studied further as this link might thus be timing sensitive. In DCMi, inflammation, injury, and heart wall stress clustered (troponin/CK-MB vs CRP/NT-proBNP, positive), consistent with the clinical association to chamber dilation in inflammatory cardiomyopathy. Coupling of ECM remodeling and heart functions was present; EF-positive relations were treated as exploratory as they are sensitive to their relative timing, stage and severity of the disease and to local tissue heterogeneities. Even though our findings are based on a small number of patients per group, the correlation analyses indicate a possible correlation between altered fibronectin fiber tension and deterioration of cardiac function. Note though that small sample sizes in Spearman rankings reduce the ability to identify true associations or give rise to perfect Spearman’s rank correlations (r = 1.00 or r = -1.00), and thus false positives. Several perfect Spearman’s rank correlations are indeed seen in our data especially for DCM (N = 6) and COVID-19 (N = 4) where we had access to small sample sizes only.

## Discussion

Intricacies of the local ECM-cell crosstalk, in synergy with the activity of ECM proteases and their inhibitors, is well balanced during homeostasis. Following viral myocarditis (MC) which imposes inflammatory-driven damage to the heart muscle, this balance gets either reestablished in a scar-free healing process, or alternatively, the severity of perturbations will ultimately determine the clinical outcome whether a tissue can heal or will undergo fibrotic transformations. For example, MMP-2, MMP-3, and TIMP-2 concentrations were related to left-ventricular ejection fraction, and MMP-3 levels correlated with longitudinal deformation, indicating that proteolytic cleavage of ECM fibers by MMPs plays important roles in the post-inflammatory re-modeling of the myocardium [57]. Therefore, enhancing our understanding of this crosstalk of resident cells with their ECM, and vice versa, may provide so-far lacking insights into the pathogenesis leading to heart failure and suggest new approaches to novel targeted therapies [[58], [59], [60], [61]]. Myocardial fibrosis is accompanied by an excessive deposition of fibrillar collagens and thereby compromises the systolic and diastolic performance [13]. While it is well established in the literature that the extent and specifics of ECM stiffening can vary depending on the severity of the MC [4], we discovered here that alterations of fibronectin fiber tension upon viral infection are inherent to the disease, already in the acute phase as well as in the scarring heart. Biopsy tissues from MC patients consistently show the appearance of loci in which fibronectin fibers that have lost their tension already in the MC group. While fibronectin fibers are maintained under high tension in the healthy human heart muscle, the staining of biopsy tissues with our well validated FnBPA5 mechanosensor [[41], [42], [43], [44], [45]] revealed the appearance of loci enriched with untensed fibronectin fibers upon viral infection, including flu and COVID-19 infections. This is a crucial observation, as stretching of ECM fibers is known to alter their display of molecular binding sites [39,46]. Other studies asking how the mechano-signature of ECM gets altered during acute infection, in fibrotic pathologies or in various cancers have revealed that the loss of fibronectin fiber tension is a mechanical hallmark of pathologically transformed ECM [42,44,45].

Already in all acute MC patients, loci of untensed fibronectin fibers in endomyocardial biopsies had emerged and spatially correlated with regions of infiltrated macrophages, as well as the viral presence in the blood. Histological analysis further indicated that fibronectin fiber tension could be lost despite the absence of any visible CMs necrosis. A significant loss of fibronectin fiber tension was seen, for example, in areas of viable cardiomyocytes (αSMA positive) in the biopsy of MC patient (#14) who was tested positive for PVB19 viral genome. Moreover, viruses are often not completely eliminated by the immune system and remain detectable throughout the lifetime, even though they do elicit a continued inflammation [1,2]. Therefore, persistent presence of viruses may not necessarily underlie the loss of fibronectin fiber tension. Our data suggest that the loss of fibronectin fiber tension precedes the dilation of the heart: a significantly positive correlation was found between creatine kinase (CK) level, an indicator of muscle damage, and untensed fibronectin fiber content in the acute MC group. This gives first hints to a possible role of cardiomyocyte injury, perhaps induced virally and/or by neutrophil infiltration, with downstream effects on the fibronectin fiber tension, or vice versa. Acute cardiac inflammation involves infiltration of neutrophils in the early phase [62,63] which sets the stage for subsequent repair of tissue damage by macrophages infiltration [64] as stimulated by neutrophil-secreted proteases [45]. In case of bacterial intestinal infections, we observed that neutrophil infiltration preceded fibronectin fiber relaxation [45]. This could suggest that the presence of neutrophils might be involved in fibronectin fiber relaxation during acute MC too. Our myocardial biopsy data showed a significant fraction of infiltrated macrophages in spatial proximity (≤5.4 µm) to untensed fibronectin fibers independent of whether fibrotic lesions had already formed, or not. This indicates that fibronectin fibers lose their tension, at least in some inflamed loci, prior to the onset of collagen fiber deposition. These observations are consistent with recent findings in COVID-19-associated myocarditis, where macrophage-driven inflammation transitioned into fibrotic ECM remodeling. Maatz et al [35] demonstrated that macrophage-enriched regions in the myocardium can evolve into fibrotic zones characterized by ECM expansion and loss of contractile elements. The detection of relaxed fibronectin fibers in both inflammatory and fibrotic contexts in our study may thus reflect a structural correlation of this inflammatory-to-fibrotic transition. Future studies using larger cohorts need to reveal though whether the loss of fibronectin fiber tension in acute MC might proceed macrophage infiltration or even steer it. Also, whether viral-mediated destruction of CMs and consequently the associated ECM remodeling, or adaptations of CMs and cardiac fibroblasts to infiltrating immune cells and thus to the inflammatory microenvironment resulted in altered fibronectin tension needs to be further evaluated.

The later myocardial inflammation phase associated with heart dilation is marked by a major decline of cardiac function: it coincides with a punctuated loss of fibronectin fiber tension often, but not always in proximity to major fibrillar collagen deposits. The loss of heart function (LV-EF and LVEDD) in dilated cardiomyopathy (DCM) and then declining in chronic inflammatory dilated cardiomyopathy (DCMi), correlated with the untensed fibronectin fiber content, with + 0.54 in DCM and only + 0.35 in DCMi. In the chronic DCMi group, a significant positive correlation was found between macrophage density in the biopsy and CK-MB, a blood marker of cardiac muscle damage arguing for the role of chronic inflammation on cardiac injury.

While suggested from our small patient numbers, further studies are needed to explore the hypothesis that fibronectin fiber relaxation in the acute inflammatory phase (MC) could potentially prime the niche for subsequent fibrotic progression as orchestrated by myofibroblasts. Our findings of the presence of αSMA-positive myofibroblasts and thick collagen I/III deposits in proximity to areas of untensed fibronectin fibers of DCM and DCMi are in line with recent studies of tumor tissues [[42], [43], [44]]. Collagen fibrillogenesis requires the presence of fibronectin fibers [36] and untensed fibronectin fibers can serve as templates to induce collagen fibrillogenesis [37]. Importantly, the onset of collagen fibrillogenesis is regulated by fibronectin fiber tension, and only untensed fibronectin fibers can serve as template [37]. In DCMi, myofibroblasts continue to be active post healing, with a positive-feedback loop resulting in continuous synthesis and assembly of collagen I and III leading to progressive cardiac fibrosis and heart failure [14,16,17]. As mentioned above, several factors can contribute to inducing the loss of fibronectin fiber tension, including proteolytic cleavage as well as the build-up of collagen fiber bundles, as myofibroblasts might switch from fibronectin fiber adhesion to anchoring themselves on the more rigid collagen fibers thereby contributing to the loss of fibronectin fiber tension [37]. In acute myocarditis, elevated CK-MB levels reflect very early necrosis; when heart geometry factors are assessed in the same window, CK-MB–LVEDD can correlate positively, compatible with early dilation. By contrast, EF is global and load-dependent and is often measured outside the troponin peak; in focal presentations EF may remain preserved despite marked biomarker release. This differs from large transmural myocardial infarction, where high troponin and low EF co-occur. As the rise of blood biomarkers typically precedes the onset of measurable cardiac functional changes, we therefore defined the MC troponin–EF signal correlation as timing-sensitive and exploratory.

To assess the physiological implications, it is important to note the following: the loss of fibronectin fiber tension does not only change its viscoelastic module but can directly tune the affinity of fibronectin’s binding partners [39]. Beyond fibronectin’s structural role, it displays various integrin-binding sites and serves as a reservoir to locally enrich signaling molecules such as growth factors and cytokines, whereby the binding of at least some of those might be regulated by the tension of fibronectin fibers. For example, tissue transglutaminase (TG2), which is highly upregulated upon ECM remodeling in other diseases, such as fibrosis and cancer, binds tighter to untensed than to stretched fibronectin fibers [65]. TG2 covalently links the TGFβ latency complex to fibrillar ECM proteins fibrillin and fibronectin and thereby plays an essential role in TGFβ activation during inflammation and fibrotic development [66]. TG2 is involved in cardiac signaling, contractility and ischemia/reperfusion injury [67]. Recently, mechanistic studies using both in vitro as well as in vivo mouse models have shown the involvement of TG2 in cardiac fibrosis through TGFβ1-induced transition of fibroblasts to myofibroblasts [68], in addition to its role in promoting the endothelial-mesenchymal transition (EndoMT) [69]. Moreover, loss of fibronectin fiber tension can lead to a release of interleukin-7 (IL-7) [70], which is much more potent in promoting immune cell maturation, T cell homeostasis and adaptive immunity, when tethered to the ECM via fibronectin-binding [39]. Circulating IL-7 was suggested to aggravate myocardial ischemia–reperfusion injury by promoting CMs apoptosis and macrophage infiltration [71]. As regions of untensed fibronectin fibers were rich in infiltrating immune cells (macrophages, T cells), suggesting that their reciprocal crosstalk might impact the immune cell niche. Furthermore, fibronectin relaxation could also mechanically tune CMs functions. In a healthy adult heart, for example, CMs express laminin-binding α_7_β_1_ integrins. However, during development and disease, the fibronectin-binding α_5_β_1_ and collagen-binding α_2_β_1_ integrins are highly expressed by fibroblasts alongside increased secretion of fibronectin and collagens [72]. Thereby, relaxation of fibronectin fibers, which are recognized by α_5_β_1_ integrins, as well as deposition of fibrillar collagen could impact the force generation and transmission of CMs in regions of major ECM remodeling [73]. Considering fibronectin’s immunomodulatory potential and tension-dependent interaction with transiently upregulated ECM enzymes, a detailed analysis of the role of fibronectin fiber tension in the progression of inflammation and deterioration of cardiac function is required. Thus, the loss of fibronectin fiber tension observed here in myocarditis could play a pivotal role in priming the myocardial niche towards fibrotic transformations. Overall, our data suggest that the loss of fibronectin fiber tension may be an intrinsic feature of ECM remodeling in myocarditis, both during acute inflammation and in post-inflammatory fibrosis. Although causality cannot be established, and sample size was limited, the consistent detection of relaxed fibronectin fibers in distinct pathological contexts highlights their potential physiological relevance. This hints to the possibility that fibronectin fiber relaxation primes cell niches for subsequent fibrotic progression. These findings support the hypothesis that ECM mechano-alterations may contribute to disease evolution and merit further mechanistic and translational investigation.

## Conclusion

Taken together, our correlative analyses suggest that a loss of fibronectin fiber tension is observed in myocarditis—both during acute inflammation and within fibrotic regions—and may be associated with indices of cardiac dysfunction. In this small, heterogeneous cohort, the observed alterations in fibronectin fiber tension are thus exploratory; larger, longitudinal studies are required to validate these associations and to assess the feasibility of in-vivo diagnostics.

Mapping the physical states of extracellular matrix (ECM) molecules—such as differentiating between stretched (healthy) and relaxed (pathological) fibronectin fibers—represents a powerful new class of “mechano-markers”. Unlike traditional diagnostics, which focus only on the presence of molecules detected by antibodies, mechanobiological signatures reveal how these molecules are mechanically altered in disease. Our data suggest that this distinction might be clinically significant: our quantification methods can distinguish distinct patient phenotypes even when they share similar diagnoses. By integrating mechanobiological signatures of ECM remodeling that map molecular physical states alongside existing immune biomarkers, clinicians may improve diagnostic precision and better predict healing and scarring outcomes. Ultimately, these innovations could transform prognosis and therapeutics for challenging inflammatory diseases like myocarditis.

### Clinical perspectives

Myocardial imaging allows for detailed assessment of cardiac anatomy, function, and tissue composition, as well as their pathological changes in various diseases. Traditionally, cardiovascular magnetic resonance (CMR) has been the primary method for imaging myocardial fibrosis. Although this study included a limited number of patient samples, it highlights the pivotal role of extracellular matrix (ECM) remodeling and its mechanobiological changes in the progression of human viral myocarditis. Our research suggests, to our knowledge in this cohort, that the tension of fibronectin fibers within the ECM is altered in inflamed human hearts and that these changes persist into the post-inflammatory fibrotic stage. This underscores the heterogeneity of the ECM in viral myocarditis, both in its composition and mechanical properties, and points to the urgent need for distinct mechanical markers to track disease progression and predict clinical outcomes. Since myocarditis diagnosis mainly relies on endomyocardial biopsies, further research into mechanobiological changes in the ECM could help identify new ex vivo markers for disease status and prognosis. Additionally, our use of a tension-sensitive peptide probe—capable of being radiolabeled and targeting diseased ECM in mice [41]—shows promise for advancing clinical imaging. Unlike conventional methods that target upregulated proteins, this approach focuses on the physical state of specific ECM fibers in vivo, representing a novel strategy for myocarditis imaging.

### Limitations of the study

The analyses in this work are based on human cardiac tissue with varying disease etiologies and sampling times, resulting in inherent heterogeneity. As patients were diagnosed upon presentation of clinical symptoms of myocarditis such that the onset of the disease is unknown, the patients could represent different stages of inflammation (as indicated by the blood C-reactive protein levels) and/or different extent of cardiac damage (troponin T levels). This could explain the high heterogeneity in immunohistochemical biopsy data, as well as in the blood parameters and cardiac function data.

While we identify spatial correlations between fibronectin fiber relaxation, immune cell infiltration, and fibrotic markers in dilated hearts, these associations are descriptive in nature. Therefore, an in-depth characterization of these phenotypes is required to learn how to best exploit mechanobiological markers for the improvement of treatment strategies and prognosis. The correlation analyses in this work should be considered exploratory and hypothesis-generating, given the small sample size and inter-individual variability. Nevertheless, finding correlations of clinical and biopsy data underlines the significance of our findings with respect to cardiac ECM remodeling and functions.

Our data also indicate that patients when clinically grouped based on histopathology and cardiac function tests (MC, DCM, DCMi and COVID-19) can display complex sub-phenotypes as suggested by our immunohistochemical stains of myocardial biopsies. However, causality cannot be inferred from static histological observations, and functional studies will be necessary to determine whether fibronectin fiber tension actively contributes to disease progression and whether it can be exploited for early clinical diagnostics or drug targeting thus needs further explorations.

## Materials and methods

### Preparation of tissues for (immune) histology and stainings

Left ventricular (LV) endomyocardial biopsies were obtained from patients upon presentation of clinical symptoms with suspected myocarditis. LV-endomyocardial biopsies were performed through the right femoral artery, where a long guiding sheath was mounted over an angulated pigtail catheter that was used as a support during the sheath positioning. The bioptome was then slowly advanced against the cardiac wall outside the sheath to collect the myocardial specimen [74]. The tissue blocks were snap frozen with liquid nitrogen and embedded in OCT. Sections of thickness 20 µm were cut from the tissue blocks and stored at −20 °C until further analysis. To cross-check our histological findings, formalin-fixed paraffin embedded (FFPE) sections used for diagnosis of myocarditis were used.

### Preparation of peptide stock

FnBPA5 (CGGGQVTTESNLVEFDEESTKGIVTGAVSDHTTVEDTK) and scrambled FnBPA5 (CGSEQEDLTGTKVDFGETIVVNEATETVTSGSTHGTKV) were commercially synthesized and photolabeled with Cy5 fluorophore. The lyophilized peptides were dissolved in ultrapure water at a concentration of 1 mg/ml and stored in −20 °C until further usage.

### Histological stainings

Histological analyses were performed on the 20 µm thick cryosections as described in Supplement section (along with Suppl. Table 4).

### Confocal and second harmonic generation (SHG) microscopy

The mounted tissue sections were imaged under the Leica SPF6 confocal microscope. The microscope was equipped with 488 nm, 543 nm and 647 nm and Multiphoton laser lines to image the samples. The LAX UI software was used to control the microscope and perform the imaging. The laser lines, laser intensity, gain and confocal pinhole were adjusted according to the image requirements. Sequential scanning mode was used to image multiple fluorescent markers in separate channels. Moreover, as tissue areas were larger than a single frame, tile scan mode was used with a default 10 % overlap to produce the stitched final image. To acquire signals from multiple slices of the tissue section Z-stack imaging was used. The fluorophores were excited with 488 nm, 543 nm and 647 nm laser lines and the emission was captured using 500 – 525 nm, 550 – 590 nm and 640–––680 nm band pass filters. For the multiphoton laser, the excitation wavelength was 910 nm for collagen and 750 nm for DAPI and the emission band pass filter 450 – 460 nm and 375 – 475 nm was used respectively. The images were then exported as stitched tile image for each channel and each Z slice for further analysis.

### Image analysis

#### Analysis of macrophage infiltration

In order to quantitatively distinguish the tissues with high macrophage-density from the rest of the tissues, an Image J macro was used. As a first step, the Z-stack images from each channel were combined to result in a single image with maximum intensity from all slices. Using an interactive feature in the macro, the total tissue area was marked manually. In order to compare multiple images with different tissue areas, the tissue area was divided into square grids of the same dimensions (50x50 pixels/ 30.27x30.27 µm). A threshold value and de-noise value was applied to remove unspecific and background signals. After applying the threshold value, the macro evaluated the mean CD68 + pixel area, mean intensity and mean integrated intensity (mean gray value x area). The normalized intensities across the tissues were given as heat maps. The mean CD68 + pixel area after thresholding was calculated for all the grids in the image. To compare the spatial distribution of macrophages, the mean CD68 + pixel per square grid area was calculated for each image and compared. Only the tissues with more than 0.2 % (mean % of CD68 + pixels per square grid area) of CD68 positive pixels were considered tissues with high macrophage-density indicating macrophage crowding.

#### Untensed fibronectin fiber and collagen fiber (SHG) pixel analyses

The content of untensed fibronectin and collagen fibers was analyzed quantitatively from the histological staining and SHG images respectively. As the total tissue area was different for each sample, the untensed fibronectin content was calculated as a ratio of area of total tissue fibronectin (stained by a polyclonal fibronectin antibody) and the collagen content as a ratio of total tissue areas. To calculate the contents, another Image J macro was used. In the macro, the total area/ fibronectin area was manually selected. The threshold or de-noise value was entered to exclude background noise. The macro then calculates the ratio of area of untensed fibronectin fibers to the total fibronectin area and ratio of collagen area to the total area. Since SHG imaging at 910 nm wavelength results in overlap with signals from the DAPI channel, the macro creates a mask for areas with overlapping DAPI and collagen signals and subtracts the DAPI area from the collagen area.

#### Spatial proximity analysis

To analyze the spatial proximity of CD68- and FnBPA5-positive pixels, a spatial proximity analysis was performed using a Matlab routine as described in [42]. The algorithm generates a merged image of CD68 and FnBPA5 channels. A 9x9 pixel matrix, which corresponds to the size of 5.4x5.4 µm^2^ was generated around each pixel. The algorithm runs over each of the pixels of one channel and searches for pixels of the other channel within the matrix. Whenever a pixel was found it was calculated as a positive value. To calculate the CD68 pixels in proximity to FnBPA5, the total number of CD68 pixels in proximity to FnBPA5 pixels was calculated and plotted as a percentage of total number of pixels for the FnBPA5 channel. Vice versa, to calculate the FnBPA5 pixels in proximity to CD68 pixels, the total number of FnBPA5 pixels in proximity to CD68 was calculated and plotted as a percentage of total number of pixels for the CD68 channel.

### Statistical analysis

Statistical analysis was performed using GraphPad Prism software. All statistical data are given as mean ± s.e.m. To compare the groups, t-tests (non-parametric), one-way ANOVA (non-parametric) and Spearman correlation analysis were performed. Significance levels were set to α = 0.05. Statistical significance is indicated by * P ≤ 0.05, ** for P ≤ 0.01, *** for P ≤ 0.001, **** for P ≤ 0.0001 and ns for P > 0.05. All values in the plots are given as Mean ± S.E.M.

### Clinical diagnostic data

Myocarditis was clinically diagnosed by established histological (Dallas criteria), immunological, and immunohistochemical criteria (presence of > 14 leucocytes/mm^2^ of the biopsy tissue area including up to 4 monocytes/mm^2^ with the presence of CD3 positive T-lymphocytes > 7 cells/mm^2^) [75]. DCM is clinically diagnosed by fractional shortening (FS; percentage shortening of left ventricular diameter during systole) < 25 % (>2 SD) or ejection fraction (EF; percentage of total amount of blood in the left ventricle pumped out during each heartbeat) < 45 % (>2 SD), and left ventricular end-diastolic diameter (LVEDD) > 117 % (>2 SD of the predicted value of 112 % corrected for age and body surface area), excluding any known cause of myocardial disease [76]. DCMi is characterized by both immune cell infiltration (with or without virus) and ventricular dilatation.

The TnT level (normal range: <14 ng/l), which is a blood marker of cardiac muscle cell damage, was highest in MC patients (359 ± 102 ng/l, mean ± s.e.m) compared to DCMi (52 ± 32 ng/l) and COVID-19 (166 ± 110 g/l) patient groups. The NTproBNP level (normal range: <97 ng/l), a marker of heart failure, was higher in DCM (5950 ± 3397 ng/l), COVID-19 (4885 ± 4828 ng/l) and DCMi (4517 ± 3445 ng/l) compared to the MC group (917 ± 364 ng/l). The CK level (normal range: <190 U/l), a marker of muscle cell damage due to hypoxia or other injury, was highest in DCMi (249 ± 132 U/l) compared to MC (184 ± 63 U/l), DCM (180 ± 64 U/l) and COVID-19 (177 ± 70 U/l) patient groups. The CK-MB level, a marker of cardiac-specific muscle cell damage (normal range: 24 U/l) were comparable across the patient groups, with MC (31 ± 11 U/l), DCMi (28 ± 8 U/l) and COVID-19 (26 ± 6 U/l). The hsCRP levels (normal range: <5 mg/l), a marker of inflammation, was highest in COVID-19 patients (234 ± 224 mg/l) compared to DCMi (21 ± 12 mg/l), MC (16 ± 5 mg/l) and DCM (11 ± 8 mg/l).

## Ethical statement

Endomyocardial biopsies were collected from patients for the primary purpose of diagnosis [[77], [78], [79]] as well as for research purposes following Ethics Committee approval (EA2/140/16) and informed consent. The investigations conform to the principles outlined in the Declaration of Helsinki [80].

## CRediT authorship contribution statement

**Krishna Chander Sridhar:** Writing – review & editing, Writing – original draft, Visualization, Investigation, Formal analysis. **Julia Mehl:** Writing – review & editing, Investigation. **Karin Klingel:** Resources. **Mario Thiele:** Software. **Sophie Van Linthout:** Writing – review & editing. **Carsten Tschöpe:** Supervision, Resources, Methodology, Funding acquisition, Conceptualization. **Georg N Duda:** Supervision, Resources, Methodology, Funding acquisition, Conceptualization. **Viola Vogel:** Writing – review & editing, Resources, Project administration, Funding acquisition, Conceptualization.

## Declaration of competing interest

The authors declare the following financial interests/personal relationships which may be considered as potential competing interests: [Viola Vogel reports financial support was provided by Einstein Foundation Berlin. Viola Vogel reports financial support was provided by Swiss National Science Foundation. Carsten Tschoepe reports financial support was provided by German Center for Cardiovascular Disease. Georg N Duda reports financial support was provided by Federal Ministry of Education and Research Berlin Office. Viola Vogel is co-founder of the ETH/PSI Start-up company Tandem Therapeutics If there are other authors, they declare that they have no known competing financial interests or personal relationships that could have appeared to influence the work reported in this paper.].

## Data Availability

Data will be made available on request.
