# Supplementary material for: Loss of fibronectin fiber tension is inherent to ECM remodeling in human myocarditis and post-inflammatory fibrosis
Source: Matrix Biol Plus. 2025 Oct 2;28:100182. doi: 10.1016/j.mbplus.2025.100182 (PMC12538048; doi:10.1016/j.mbplus.2025.100182)
Supplement: Supplementary Data 1 [file mmc1.docx]

**Supplementary Material**

**Supplementary Figures:**

[**Figure S1: Macrophage infiltration in inflamed myocardial tissues**](#bookmark=id.qc69ommtmcc0)

[**Figure S2: T cell infiltration in inflamed myocardial tissues**](#bookmark=id.n4fprw35s5lj)

[**Figure S3: Tension loss of fibronectin fibers in inflamed myocardial tissues shown of all patients across patient groups**](#bookmark=id.yakk638jy7gy)

[**Figure S4: Extended loci of fibronectin fiber relaxation in myocardial tissues as visualized for all patients**](#bookmark=id.fg3ja2v05pfi) [**Figure S5: Collagen fiber deposition in inflamed myocardial tissues shown for all patients across patient groups**](#bookmark=id.3jld3k8b38r9)

[**Figure S6: Loci of fibronectin fiber relaxation and MMPs shown for representative patients**](#bookmark=id.qdf88e82l3kj)

[**Figure S7: Fibronectin fiber relaxation and collagen fiber deposition in inflamed myocardial tissues shown for representative patients**](#bookmark=id.iv7d18w7nkh7)

[**Figure S8: Alpha smooth muscle actin positive myofibroblasts colocalize with loci enriched in untensed fibronectin fibers and collagen fiber deposits**](#bookmark=id.bdmkig3vodnd)

**Supplementary Tables:**

[**Table 1: List of patients together with their clinical diagnosis**](#bookmark=id.k9812jbo0q66)

[**Table 2: Clinically relevant correlations from Figure 7 in the main text**](#bookmark=id.ol6iuztb6eyn)

[**Table 3: P values for correlation matrix (Figure 7 a-d)**](#bookmark=id.oodyebalkrvh)

[**Table 4: Staining Procedure, List of markers and antibodies used**](#bookmark=id.a808djizebk0) [**Table 5: Comparison of clinical and experimental parameters in macrophage crowded vs macrophage non-crowded myocardial tissues across patient groups (MC, DCM, DCMi and COVID-19)**](#bookmark=id.qzu92yuwnfnv)

**Supplement to Fig. 1a in the main manuscript**


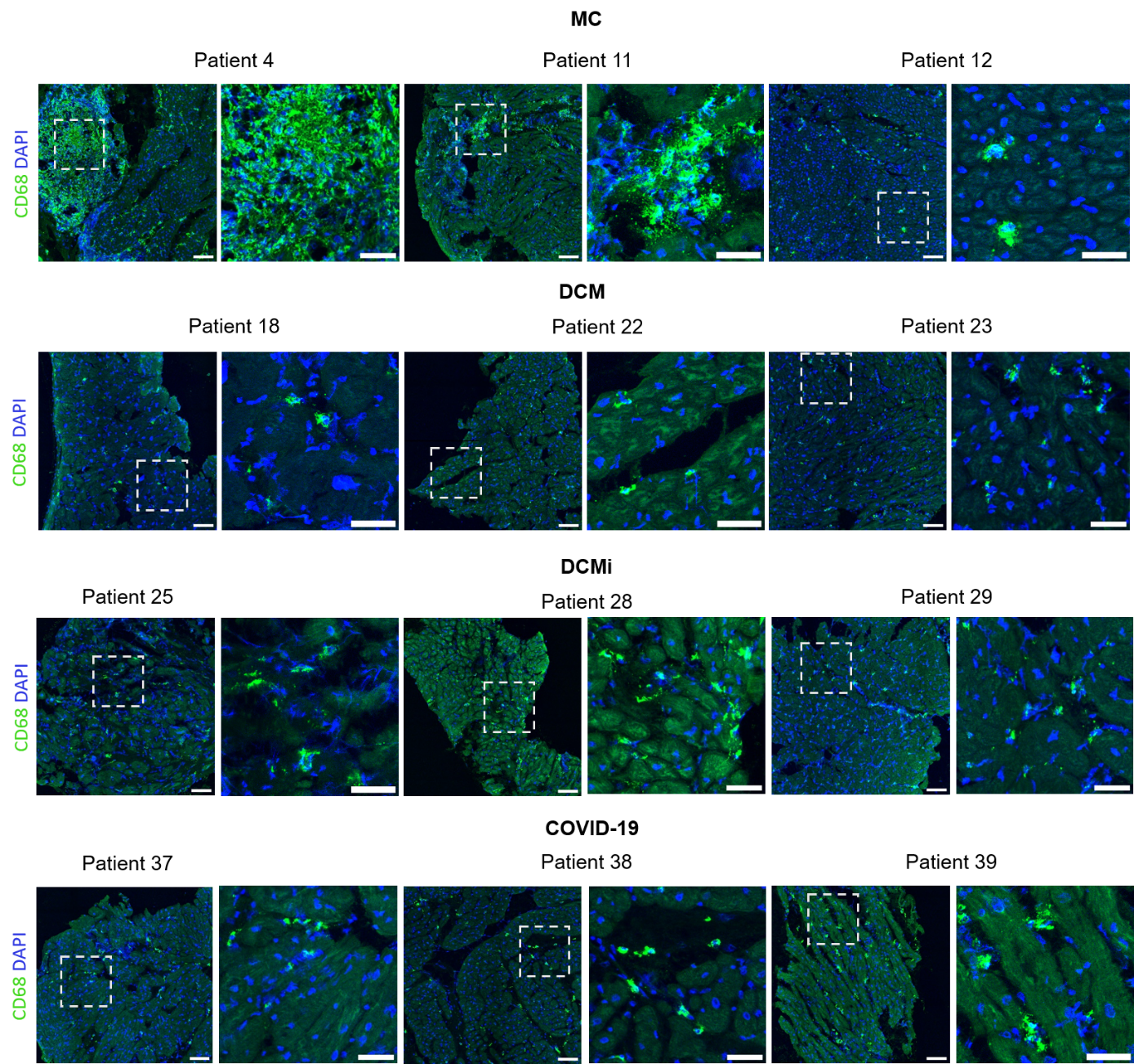


**Figure S1: Macrophage infiltration in inflamed myocardial tissues.** Immunohistochemistry (IHC) images showing CD68^+^ macrophages infiltration and spatial distribution in patient tissues across acute myocarditis (MC), dilated cardiomyopathy (DCM), inflammatory dilated cardiomyopathy (DCMi) and COVID-19, N=39, Scale bars: Overview images: 100 µm (left), corresponding enlarged images (right): 50 µm. The dotted squares indicate ROI that are enlarged beside the image for each of the patients.

**Supplement to Fig. 1 c in the main manuscript**


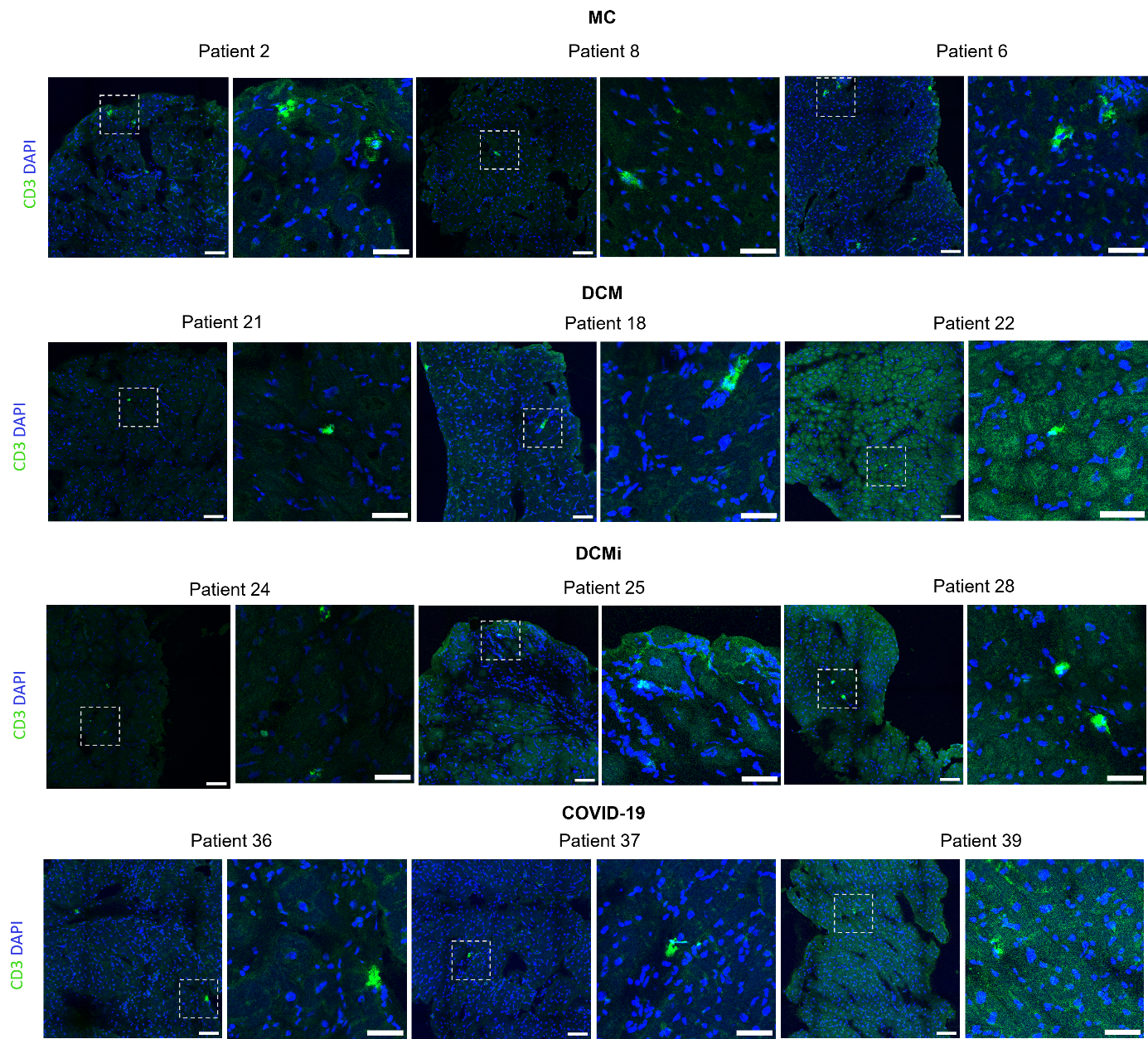


**Figure S2: T cell infiltration in inflamed myocardial tissues.** IHC images show CD3^+^ T cell infiltration in patient tissues across the patient groups, total individual patient samples, N=37, Scale bars: Overview images: 100 µm; enlarged images: 50 µm. The dotted squares indicate ROI that are enlarged beside the image for each of the patients.

**Supplement to Fig. 3 in the main manuscript**


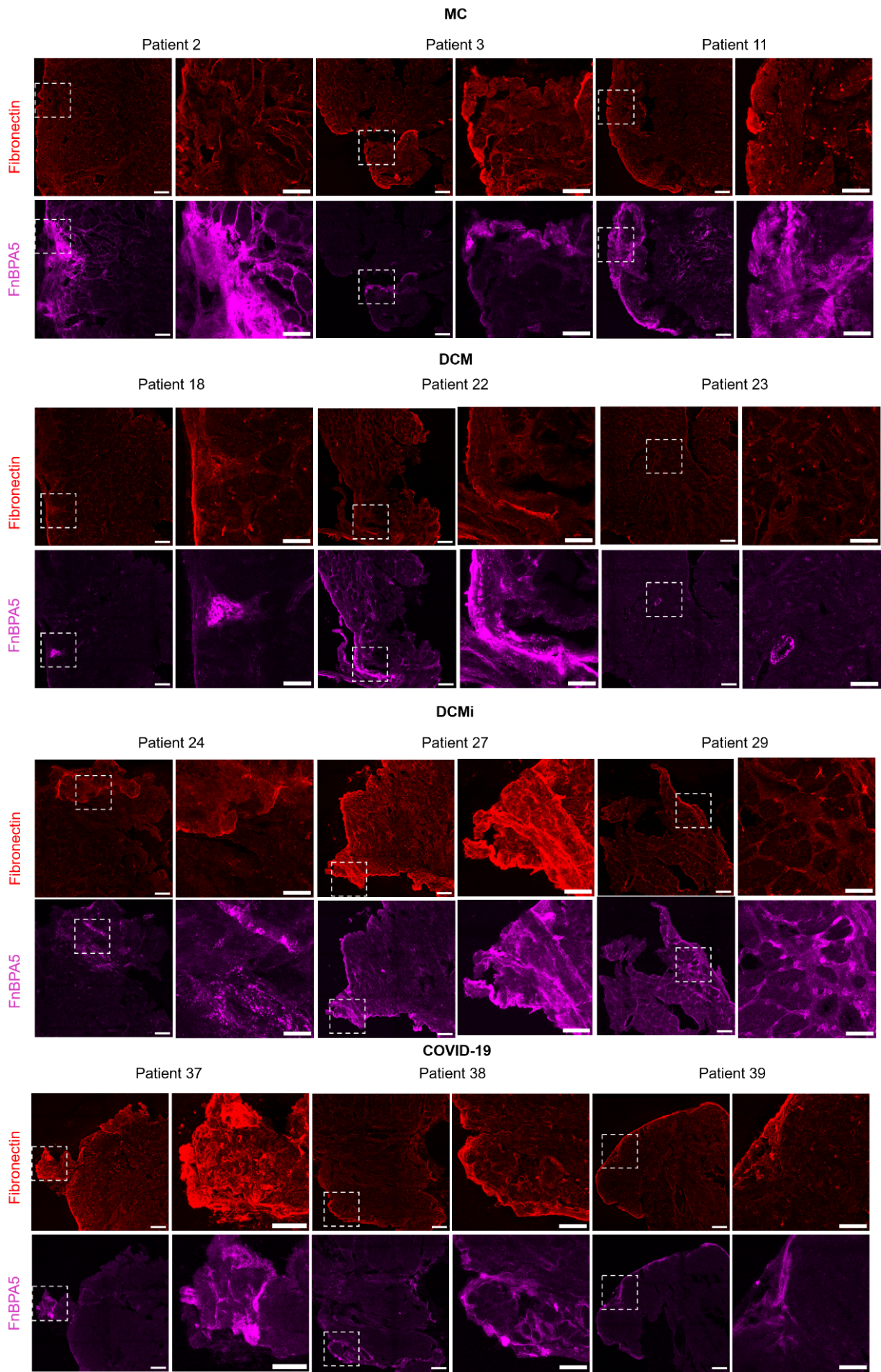


**Figure S3: Tension loss of fibronectin fibers in inflamed myocardial tissues shown for all patients across patient groups.** IHC images show total fibronectin (above; stained using polyclonal fibronectin antibody) and relaxed fibronectin fibers (below; stained using the Cy5-FnBPA5 peptide probe) indicating sites of major extracellular matrix remodeling in patient tissues across the patient groups, N=39, Scale bars: Overview images: 100 µm, Enlarged images: 50 µm. The dotted squares indicate ROI that are enlarged beside the image for each of the patients.

**Supplement to Fig. 3 in the main manuscript**


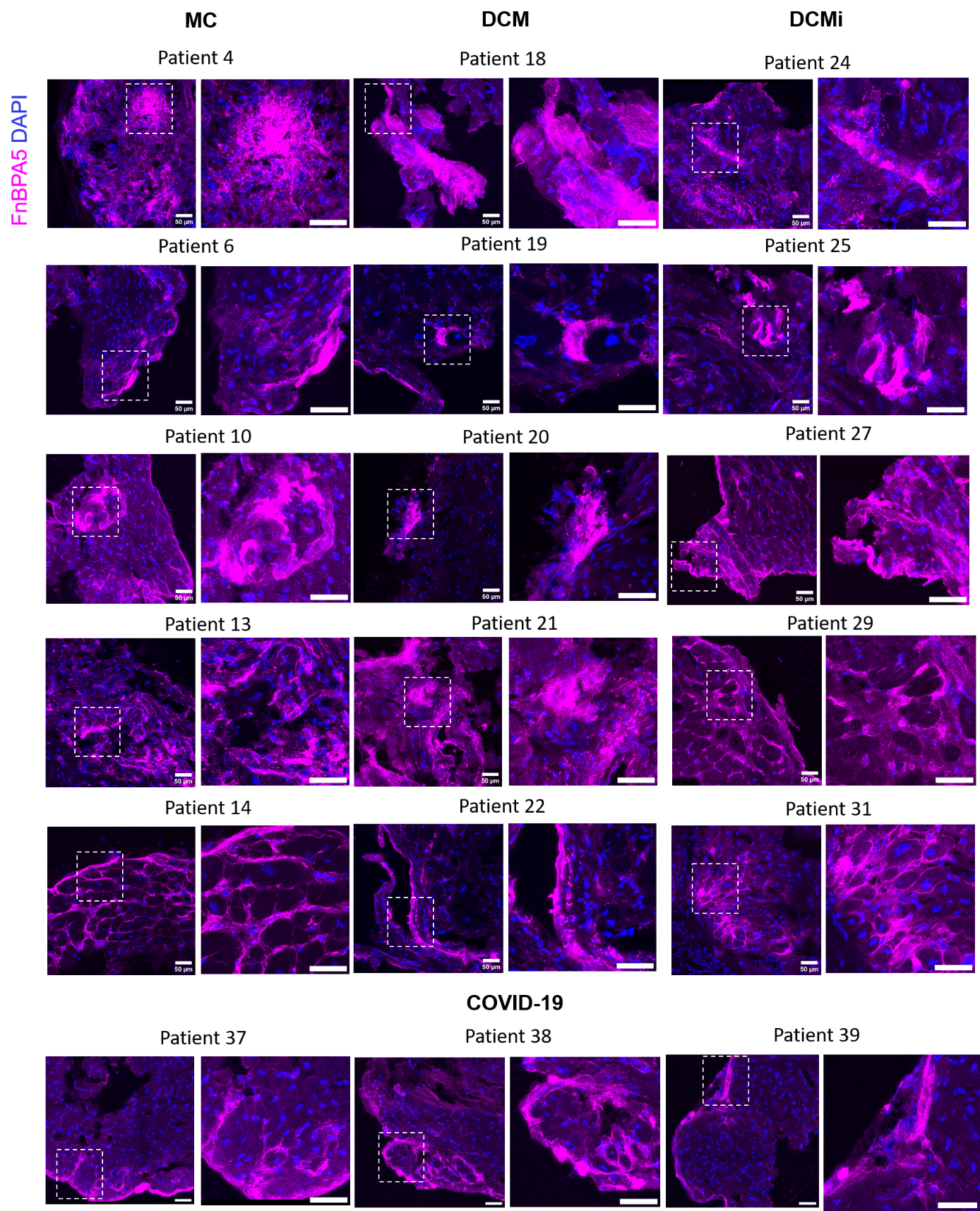


**Figure S4: Extended loci of fibronectin fiber tension loss in myocardial tissues as visualized for all patients. a)** IHC images show relaxed fibronectin fibers (Magenta) within tissue areas characterized by the presence of nuclei (blue), N=29 (MC-12, DCM-5, DCMi-8, COVID-19-4, together with zoom-in images of ROI. **b)** IHC images showing different patterns of relaxed Fn fibers – Fn fiber relaxation in interstitial areas around the cells, fibronectin relaxation in areas of tissue remodeling and fibronectin relaxation. The dotted squares indicate ROI that are enlarged beside the image for each of the patients. Scale bars: 50 µm. The dotted squares show enlarged areas.

**Supplement to Fig. 4 in the main manuscript**


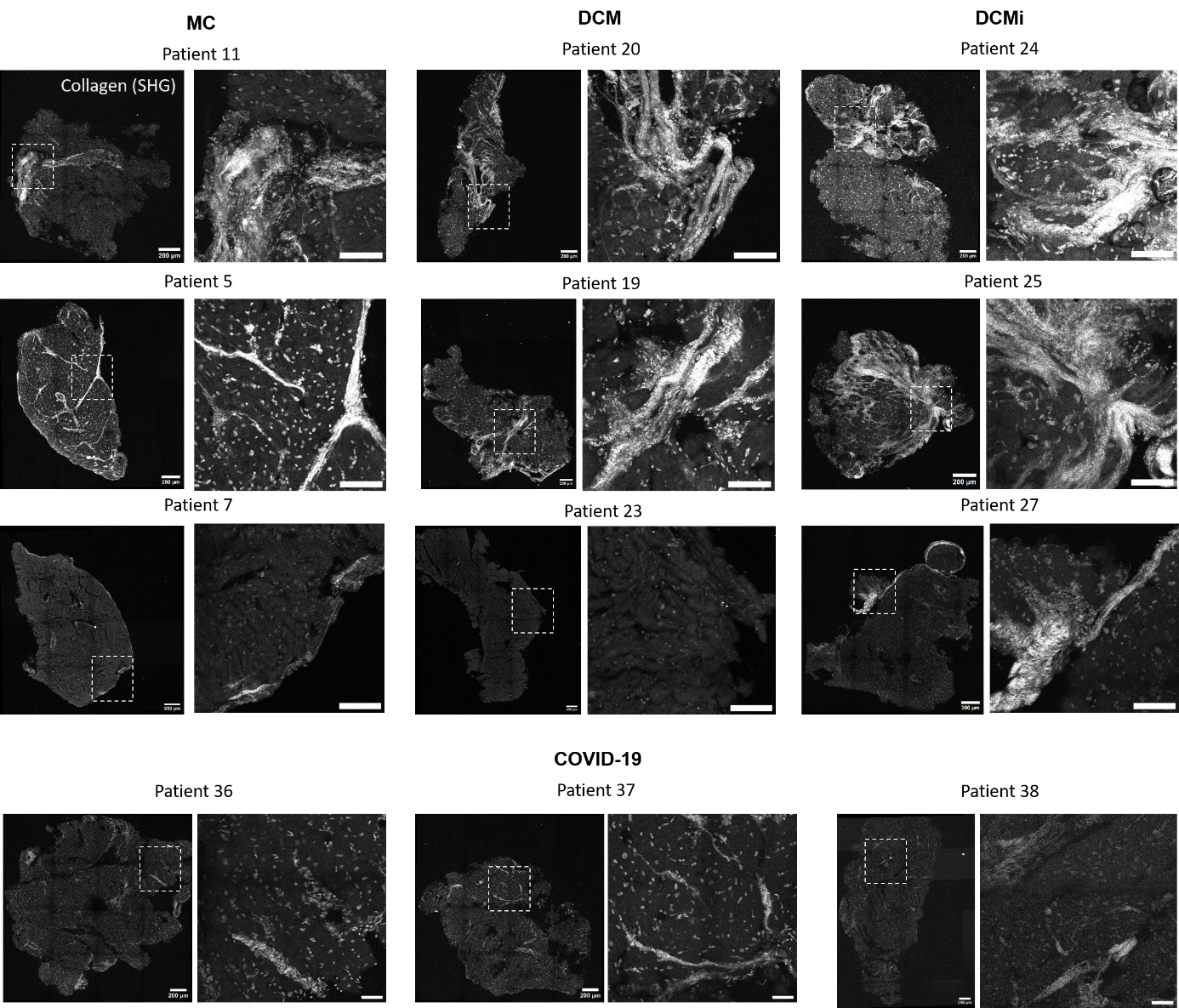


**Figure S5: Fibrillar collagen deposits as probed by SHG in inflamed myocardial tissues shown for all patients across patient groups.** Second harmonic generation (SHG) microscopy images showing thick collagen fiber bundles indicating fibrotic pathologies in tissues across the patient groups, N=35. It is not known though whether these fibrotic lesions were preexisting. Scale bars: Overview images (left): 100 µm and corresponding enlarged images (right): 50 µm. The dotted squares indicate ROI that are enlarged beside the image for each of the patients.

**Supplement to Fig. 5 and Fig. 6 in the main manuscript**

**
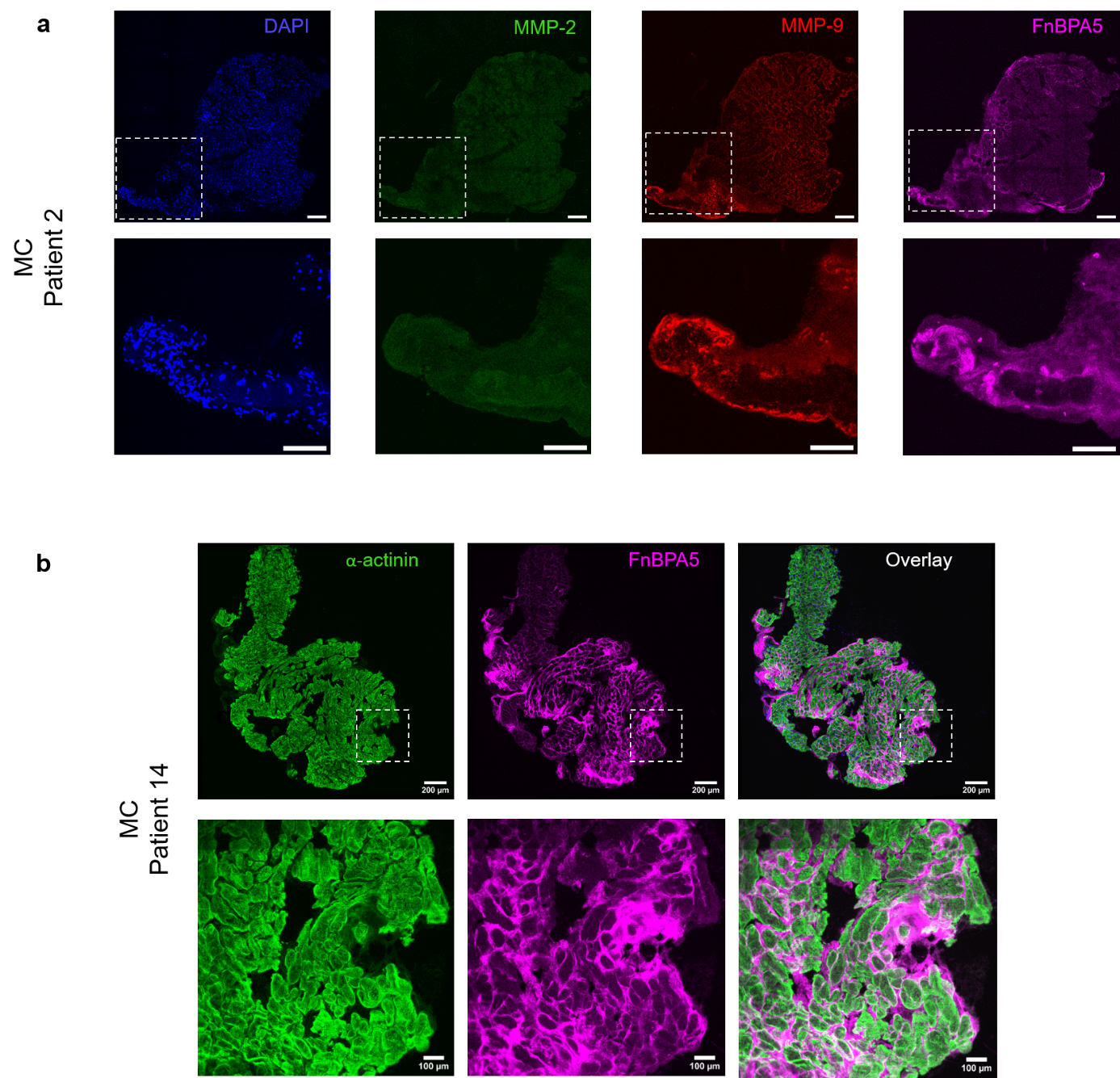
**

**Figure S6: Loci of fibronectin fiber tension loss and MMPs shown for representative patients. a)** IHC images show MMP-9 (red) proximal to relaxed fibronectin fibers (marked by FnBPA5 peptide binding) (N=2, MC-2) **b)** IHC images show sarcomeric alpha actinin indicating viable cardiomyocytes and relaxed fibronectin fibers in an acute myocarditis tissue with PVB19 viral genome detected in the endomyocardial biopsy (N=1). Scale bars: Overview images (above): 200 µm and corresponding enlarged images (below): 100 µm. The dotted squares mark ROI and are shown below.

**Supplement to Fig. 6 in the main manuscript**


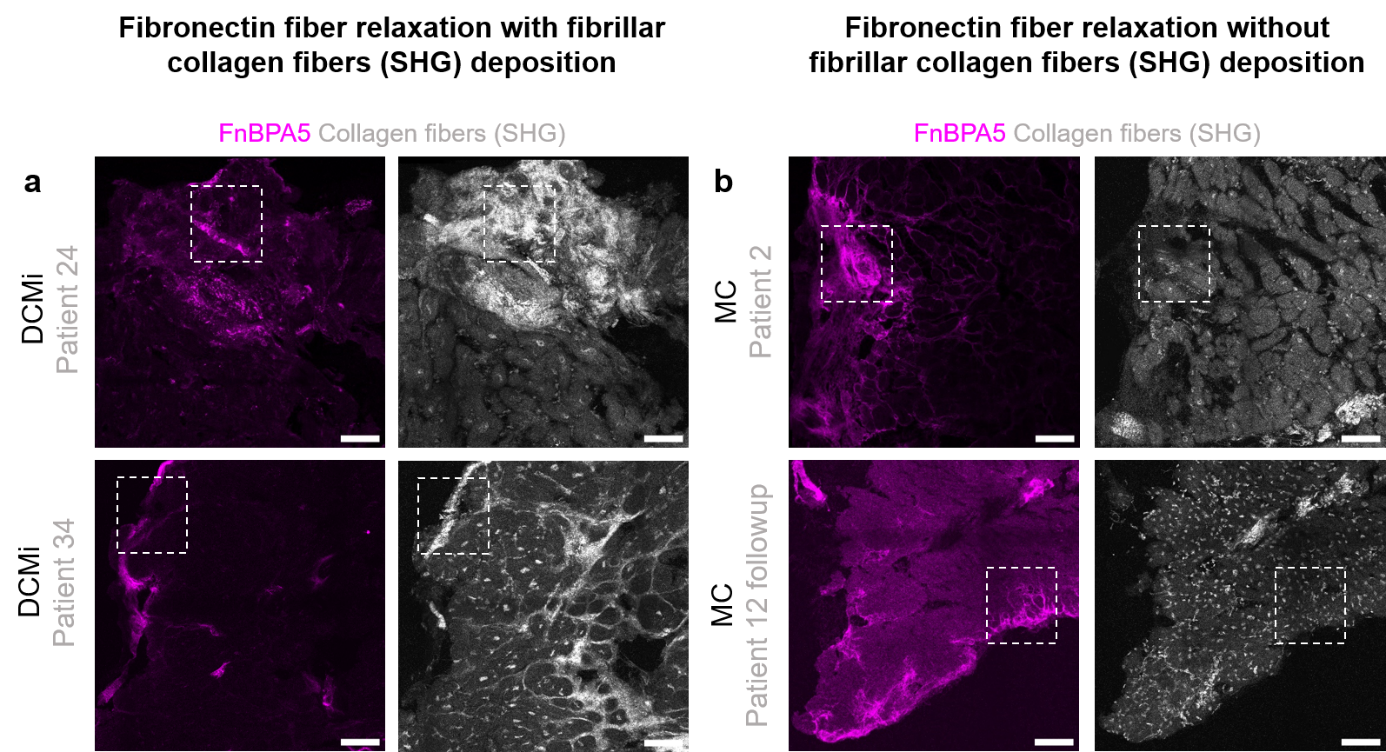


**Figure S7: Fibronectin fiber relaxation and fibrillar collagen fibers deposition as probed by SHG in inflamed myocardial tissues shown for representative patients. a)** IHC and SHG images showing fibronectin fibers that have lost their tension (marked by FnBPA5 peptide binding) and collagen fiber bundles present at same areas of the tissues as found in N=12 samples and shown here for the patients MC-4, DCM-4, DCMi-4. **b)** IHC and SHG images show the presence of relaxed fibronectin fibers in the absence of fibrillar collagen deposits in the tissues as found in N=6 samples and shown here for the patients MC-3, DCMi-3. Scale bars: 100 µm. The dotted squares show areas of significant FnBPA5 staining and the presence (left) or absence (right) of fibrillar collagen deposits (SHG).

**Supplement to Fig. 6 in the main manuscript**

**
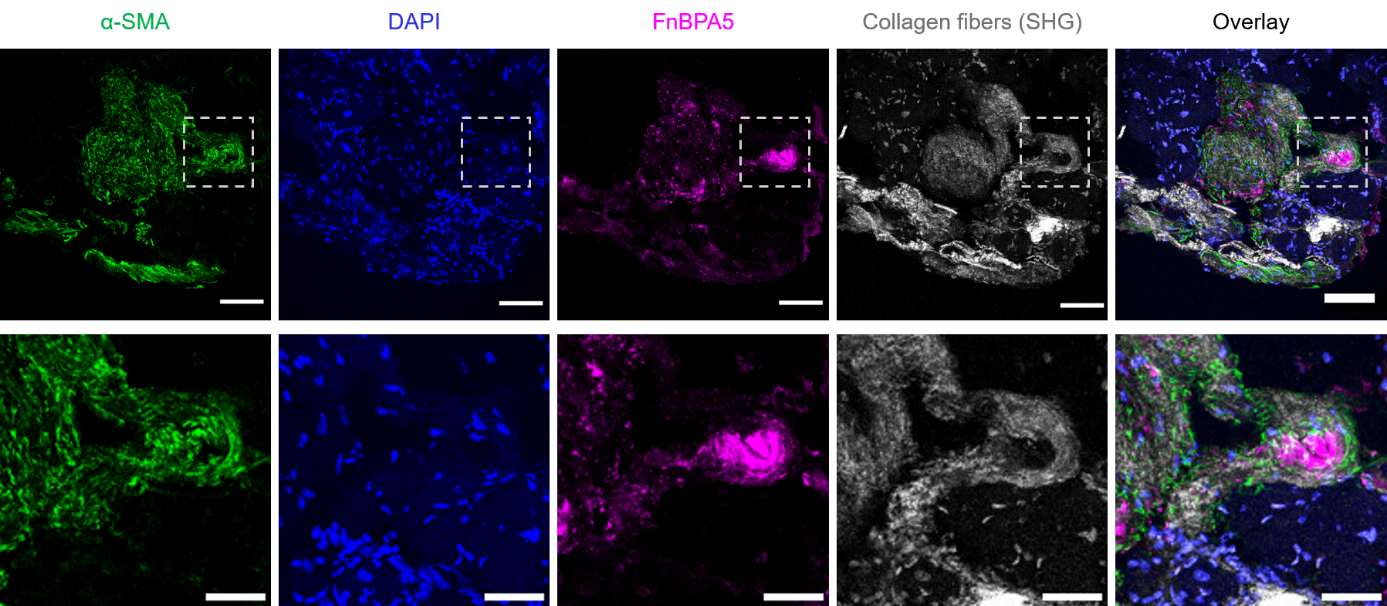
**

**Figure S8: Alpha smooth muscle actin (α−SMA) positive myofibroblasts colocalize with loci enriched in untensed fibronectin fibers and collagen fiber deposits.** IHC and SHG images show α−SMA positive myofibroblasts (green) in loci characterized by major ECM remodeling rich in untensed fibronectin fibers (magenta) and collagen fiber bundles (grey) in a follow-up biopsy of a patient diagnosed with DCMi (above) and corresponding enlarged images of areas marked in the above images (below). Scale Bars: 50 µm (above) and 20 µm (below).

**Table 1: List of patients together with their clinical diagnosis, as grouped into acute MC (red), DCM (blue), DCMi (yellow) and COVID-19 (green) patients**

| **Patient#** | **Age** | **Clinical diagnosis** | **Histological analysis (EMB)** | **Viral status (EMB)** | **Viral status (peripheral blood)** | **CMs necrosis/ caliber** | **Fibrotic features** |
| --- | --- | --- | --- | --- | --- | --- | --- |
| 1 | 22 | Myocarditis (acute) | Eosinophilic granulocytes found | - | - | Recent CMs necrosis | - |
| 2 | 50 | Myocarditis (acute) |  | PVB19 (285 copies/µg) | - | - | - |
| 3 | 46 | Myocarditis (acute) | Chronic lymphocytic myocarditis | - | - | - | Moderate diffuse interstitial fibrosis |
| 4 | 39 | Myocarditis (acute) | Sarcoidosis, lymphocytic inflammatory reaction | Virus+ PVB19 (6400 copies/µg),  Virus- PVB19 (6400 copies/µg) | - | - | Low grade perivascular accentuated interstitial |
| 5 | 25 | Myocarditis (acute) | - | - | - | - | Mild diffuse interstitial |
| 6 | 49 | Myocarditis (acute) | - | - | HHV7 virus | - | Mild diffuse interstitial |
| 7 | 37 | Myocarditis (acute) | - | - | - | - | Moderate diffuse and focal interstitial |
| 8 | 26 | Myocarditis (acute) | - | - | EBV in low copy numbers | - | Low grade diffuse interstitial |
| 9 | 53 | Myocarditis (acute) | Chronic active lymphocytic MC | - | - | - | Moderate diffuse interstitial |
| 10 | 65 | Myocarditis (acute) | Chronic lymphocytic MC, healing | - | EBV in low copy numbers | - | Moderate diffuse as well as focal partly perivascular accentuated interstitial |
| 11 | 28 | Myocarditis (acute) | Chronic lymphocytic myocarditis | PVB19 (9 copies/µg) | HCMV in low copy numbers | - | Moderate diffuse interstitial |
| 12 | 24 | Myocarditis (acute) | Beginning of cardiac illness after flu like infection, subacute lymphocytic MC | - | - | - | mild interstitial fibrosis and interstitial edema |
| 13 | 25 | Myocarditis (acute) | - | - | - | Small CMs necrosis in some places, degeneratively changed CMs | mild diffuse interstitial |
| 14 | 20 | Myocarditis (acute) | Focal giant cell myocarditis | PVB19 (485 copies/µg),  Giant cell MC in initial biopsy (?) | - | - | Moderate interstitial |
| 15 | 43 | Myocarditis (acute) | Focal acute lymphocytic myocarditis with low degree of healing | - | - | Fresh CMs necrosis in one place | Mild diffuse interstitial |
| 16 | 67 | Myocarditis (acute) | Low grade lymphocytic inflammatory reaction | - | - | Significant fluctuations in CMs caliber | Clear diffuse, focal interstitial |
| 17 | 45 | Myocarditis (acute) | Low grade lymphocytic inflammatory reaction | - | EBV in low copy numbers | fluctuations in CMs caliber, degeneratively changed CMs | Pronounced diffuse interstitial |
| 18 | 67 | Dilated cardiomyopathy (DCM) | - | - | - | Sometimes CMs fluctuation in caliber | Moderate diffuse interstitial |
| 19 | 59 | Dilated cardiomyopathy (DCM) | - | - | EBV in low copy numbers | CMs hypertrophied, degeneratively modified CM fibers | Partially clear interstitial |
| 20 | 59 | Dilated cardiomyopathy (DCM) | - | - | - | Some degenerative muscle fibers | Moderate diffuse interstitial |
| 21 | 29 | Dilated cardiomyopathy (DCM) | - | - | - | CMs fluctuations in caliber sometimes | Moderate focal and diffuse interstitial |
| 22 | 33 | Dilated cardiomyopathy (DCM) | - | - | - | Some CMs hypertrophy, degenerative fibers | Moderate diffuse interstitial |
| 23 | 50 | Dilated cardiomyopathy (DCM) | - | - | - | Slight fluctuations in CMs caliber in some places | Moderate diffuse interstitial and focal perivascular accentuated |
| 24 | 61 | Inflammatory dilated cardiomyopathy (DCMi) | - | - | - | Fluctuation in caliber, hypertrophied muscle fibers, degeneratively modified CMs | Extensive diffuse Interstitial |
| 25 | 54 | Inflammatory dilated cardiomyopathy (DCMi) | - | - | - | - | - |
| 26 | 63 | Inflammatory dilated cardiomyopathy (DCMi) | First diagnosis 4 y ago, lymphocytic inflammatory reaction | - | - | Some CMs hypertrophied and degeneratively changed | Moderate diffuse interstitial |
| 27 | 59 | Inflammatory dilated cardiomyopathy (DCMi) | Small vessel disease, mild lymphocytic inflammatory reaction | - | EBV in low copy numbers | CMs fluctuation in caliber | Moderate diffuse partly perivascular |
| 28 | 67 | Inflammatory dilated cardiomyopathy (DCMi) | Low grade lymphocytic inflammatory reaction | - | EBV in low copy numbers | CMs fluctuation in caliber | Moderate diffuse, sometimes perivascular |
| 29 | 53 | Inflammatory dilated cardiomyopathy (DCMi) | chronic myocardial damage | PVB19 (346 copies/µg) | EBV in low copy numbers | Fluctuation in caliber, hypertrophied muscle fibers, degeneratively modified CMs | Moderate diffuse interstitial, focal perivascular accentuated |
| 30 | 50 | Inflammatory dilated cardiomyopathy (DCMi) | Toxic cardiomyopathy, moderate lymphocytic inflammatory reaction | - | EBV in low copy numbers | CMs fluctuation in caliber | Moderate focal, partially perivascular |
| 31 | 49 | Inflammatory dilated cardiomyopathy (DCMi) | Moderate chronic active lymphocytic MC, healing | - | - | - | Mild diffuse interstitial |
| 32 | 38 | Inflammatory dilated cardiomyopathy (DCMi) | DCM after COVID-19 | - | EBV in low copy numbers | - | Moderate diffuse interstitial |
| 33 | 42 | Inflammatory dilated cardiomyopathy (DCMi) | Moderate lymphocytic reaction | - | - | Fluctuation in CMs caliber, hypertrophied and degenerative CMs | Moderate diffuse focal and perivascular accentuated |
| 34 | 57 | Inflammatory dilated cardiomyopathy (DCMi) | Chronic lymphocytic myocarditis | - | - | Reduced caliber of CMs | Moderate diffuse and partially perivascular |
| 35 | 57 | Inflammatory dilated cardiomyopathy (DCMi) | Onset of cardiac disease 1.5 y ago, low grade lymphocytic inflammatory reaction | - | - | Fluctuations in CMs caliber, hypertrophy and degenerated CMs | Moderate diffuse focal and partially perivascular accentuated |
| 36 | 70 | Myocarditis during/after COVID-19 | COVID-19+ week ago, urosepsis, sub-acute lymphocytic MC | - |  | Some CMs degenerative, hypertrophied CMs | Clear focal and perivascular |
| 37 | 31 | Myocarditis during/after COVID-19 | Past myocarditis with focal healing | - | EBV in low copy numbers | Some places hypertrophy, degenerated CMs | Moderate focal and sometimes diffuse interstitial |
| 38 | 32 | Myocarditis during/after COVID-19 | - | - | - | - | Clear, diffuse, partially reticular interstitial |
| 39 | 68 | Myocarditis during/after COVID-19 | - | - | - | Hypertrophy, degenerated CMs | Partially clear diffuse and focal interstitial |

**Table 2: Clinical assessment of relevant correlations from Figure 7**

| **Group** | **Variable 1** | **Variable 2** | **r (Spearman)** | **Rationale (clinical/biological)** | **Clinical anchor** |
| --- | --- | --- | --- | --- | --- |
| **MC** | CD68 in proximity to FnBPA5 | Relaxed Fn content | 0.9 | Plausible |  |
| **MC** | TropT peak | LV-Ejection fraction | 0.85 | Exploratory; Timing-sensitive (MC). | EF often assessed after biomarker peak; focal injury can preserve EF. |
| **MC** | CK-MP peak | CK peak | 0.75 | Plausible |  |
| **MC** | TropT peak | CK peak | 0.57 | Plausible |  |
| **MC** | Relaxed Fn content | CK peak | 0.54 | Plausible |  |
| **MC** | CD68 in proximity to FnBPA5 | CK-MB peak | -0.5 | Exploratory; Timing-sensitive (MC). | Proximity/tension vs acute necrosis may be anti-phase. |
| **MC** | Macrophage density | NTproBNP peak | -0.52 | Plausible |  |
| **MC** | TropT Peak | LV-End diastolic diameter | -0.68 | Exploratory; Timing-sensitive (MC). | Timing and small-n leverage can invert sign. |
| **MC** | CD68 in proximity to FnBPA5 | CRP peak | -0.73 | Exploratory; Timing-sensitive (MC). | Tension readout may decouple from bulk inflammation. |
| **MC** | Macrophage density | TropT Peak | -0.88 | Unexpected Timing-sensitive (MC). | Injury vs macrophage density should co-vary in active MC. |
| **DCM** | Collagen content | CRP peak | 0.95 | Plausible |  |
| **DCM** | FnBPA5 in proximity to CD68 | CRP peak | 0.95 | Plausible |  |
| **DCM** | Relaxed Fn content | CD68-FnBPA5 proximity | 0.94 | Plausible |  |
| **DCM** | CD68 in proximity to FnBPA5 | NTproBNP peak | 0.8 | Plausible |  |
| **DCM** | CD68 in proximity to FnBPA5 | Collagen content | 0.66 | Plausible | Proximity/tension tracks fibrosis. |
| **DCM** | Relaxed Fn content | Collagen content | 0.56 | Plausible |  |
| **DCM** | Macrophage density | CD3 count | 0.55 | Plausible |  |
| **DCM** | Collagen content | CK peak | 0.5 | Plausible |  |
| **DCM** | CD68 in proximity to FnBPA5 | CK peak | 0.5 | Plausible |  |
| **DCM** | NTproBNP peak | CRP peak | 0.5 | Plausible |  |
| **DCM** | CRP peak | LV-End diastolic diameter | -0.5 | Plausible |  |
| **DCM** | CD68 count | CRP peak | -0.5 | Plausible |  |
| **DCM** | Macrophage density | CK peak | -0.5 | Plausible |  |
| **DCM** | Relaxed Fn content | CK peak | -0.5 | Plausible |  |
| **DCM** | Relaxed Fn content | LV-End diastolic diameter | -0.5 | Plausible |  |
| **DCM** | Relaxed Fn content | CD68 count | -0.56 | Plausible |  |
| **DCM** | CD68 in proximity to FnBPA5 | CD68 count | -0.56 | Plausible |  |
| **DCM** | Collagen content | CD68 count | -0.56 | Plausible |  |
| **DCM** | Relaxed Fn content | CD3 count | -0.62 | Plausible |  |
| **DCM** | Collagen content | CD3 count | -0.62 | Plausible |  |
| **DCM** | NTproBNP peak | CD3 count | -0.63 | Plausible |  |
| **DCM** | CD68 in proximity to FnBPA5 | CD3 count | -0.77 | Plausible |  |
| **DCM** | CD68 in proximity to FnBPA5 | LV-End diastolic diameter | -0.8 | Plausible |  |
| **DCM** | NTproBNP peak | CD68 count | -0.8 | Plausible |  |
| **DCM** | NTproBNP peak | LV-Ejection fraction | -0.8 | Plausible |  |
| **DCM** | CK peak | CD3 count | -0.87 | Plausible |  |
| **DCM** | CD68 in proximity to FnBPA5 | LV-Ejection fraction | -0.94 | Plausible |  |
| **DCMi** | Macrophage density | CK-MB peak | 0.97 | Plausible |  |
| **DCMi** | Macrophage density | LV-Ejection fraction | 0.73 | Plausible |  |
| **DCMi** | CK-MB peak | CK peak | 0.7 | Plausible |  |
| **DCMi** | CK-MB peak | LV-Ejection fraction | 0.6 | Exploratory Timing-sensitive (MC). | Timing/stage mix can yield preserved EF despite injury. |
| **DCMi** | CRP peak | CK peak | 0.59 | Plausible |  |
| **DCMi** | TropT peak | CK-MB peak | 0.56 | Plausible |  |
| **DCMi** | CD3 count | CD68 count | 0.53 | Plausible |  |
| **DCMi** | CD68 in proximity to FnBPA5 | FnBPA5-CD68 proximity | 0.53 | Plausible |  |
| **DCMi** | Relaxed Fn content | LV-End diastolic diameter | 0.52 | Plausible |  |
| **DCMi** | Collagen content | CRP peak | 0.51 | Plausible |  |
| **DCMi** | NTproBNP peak | CRP peak | 0.5 | Plausible |  |
| **DCMi** | LV-Ejection fraction | LV-End diastolic diameter | -0.5 | Plausible |  |
| **DCMi** | Relaxed Fn content | NTproBNP peak | -0.51 | Plausible |  |
| **DCMi** | CD3 count | LV-End diastolic diameter | -0.54 | Plausible |  |
| **DCMi** | Collagen content | CK-MB peak | -0.6 | Exploratory | Acute injury higher in less fibrotic hearts (stage effect). |
| **DCMi** | Relaxed Fn content | TropT peak | -0.67 | Plausible |  |
| **DCMi** | Relaxed Fn content | LV-End diastolic diameter | 0.95 | Plausible |  |
| **DCMi** | CD68 in proximity to FnBPA5 | LV-End diastolic diameter | 0.95 | Plausible |  |
| **DCMi** | CK peak | LV-End diastolic diameter | 0.95 | Plausible |  |
| **DCMi** | CD68 in proximity to FnBPA5 | CK peak | 0.8 | Plausible |  |
| **DCMi** | FnBPA5 in proximity to CD68 | Macrophage density | 0.8 | Plausible |  |
| **DCMi** | Macrophage density | CD68 count | 0.8 | Plausible |  |
| **DCMi** | CD3 count | CD68 count | 0.8 | Plausible |  |
| **DCMi** | Relaxed Fn content | CK peak | 0.8 | Plausible |  |
| **DCMi** | Macrophage density | CK peak | 0.8 | Plausible |  |
| **DCMi** | CD3 count | LV-Ejection fraction | 0.77 | Plausible |  |
| **DCMi** | Collagen content | LV-Ejection fraction | 0.77 | Plausible |  |
| **DCMi** | Macrophage density | LV-End diastolic diameter | 0.63 | Plausible |  |
| **DCMi** | Relaxed Fn content | Collagen content | 0.6 | Plausible |  |
| **DCMi** | CD68 in proximity to FnBPA5 | Collagen content | 0.6 | Plausible |  |
| **DCMi** | FnBPA5 in proximity to CD68 | CK peak | 0.6 | Plausible |  |
| **DCMi** | FnBPA5 in proximity to CD68 | NTproBNP peak | 0.5 | Plausible |  |
| **DCMi** | collagen content | NTproBNP peak | 0.5 | Plausible |  |
| **DCMi** | NTproBNP peak | CD68 count | 0.5 | Plausible |  |
| **DCMi** | NTproBNP peak | CD3 count | 0.5 | Plausible |  |
| **DCMi** | FnBPA5 in proximity to CD68 | LV-Ejection fraction | -0.77 | Plausible |  |
| **DCMi** | FnBPA5 in proximity to CD68 | Collagen content | -0.8 | Plausible |  |

**Table 3: P values for correlation matrix (Figure 7 a-d in the main manuscript)**

**MC (N=17)**

**
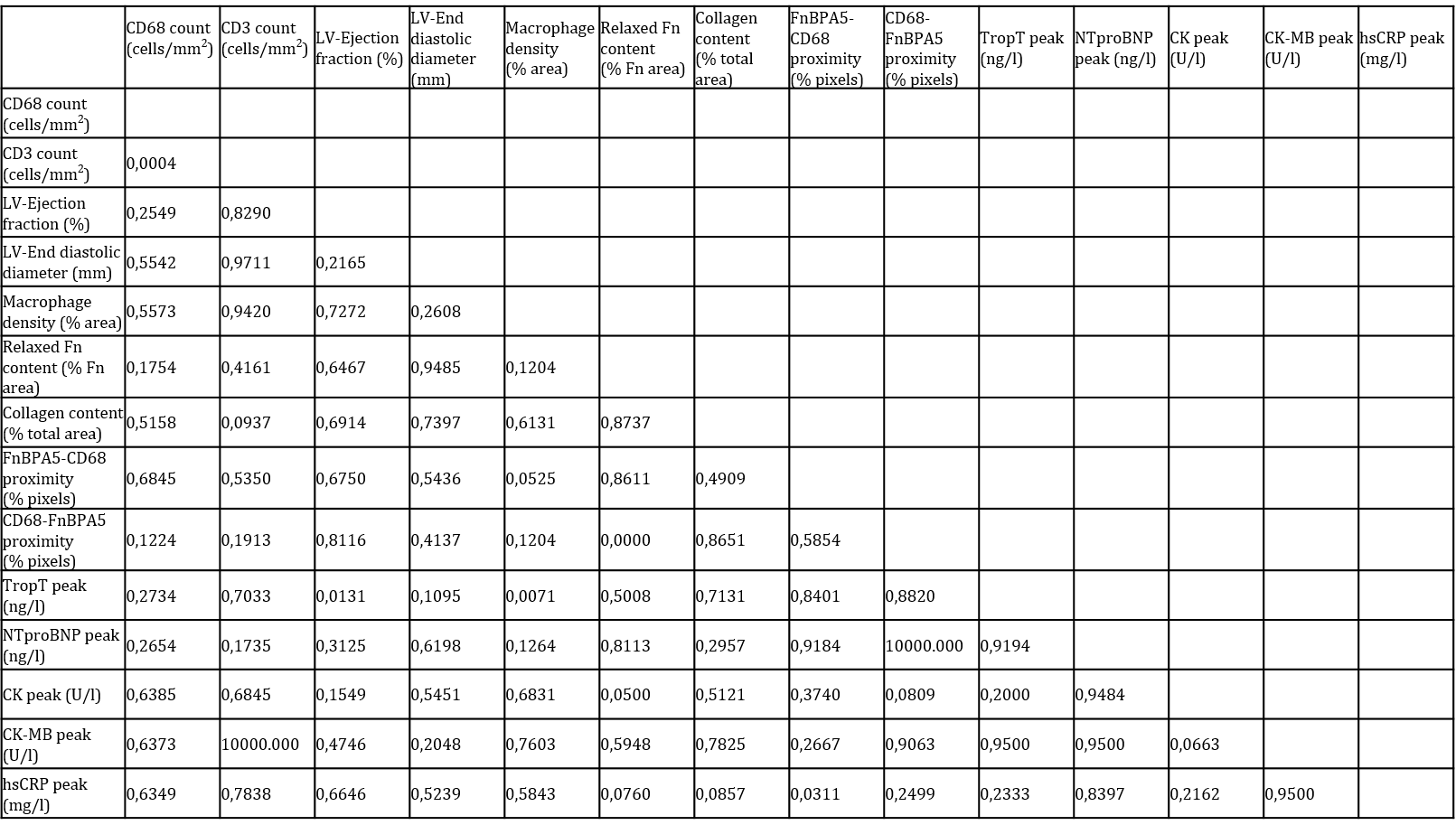
**

**DCM (N=6):**

**
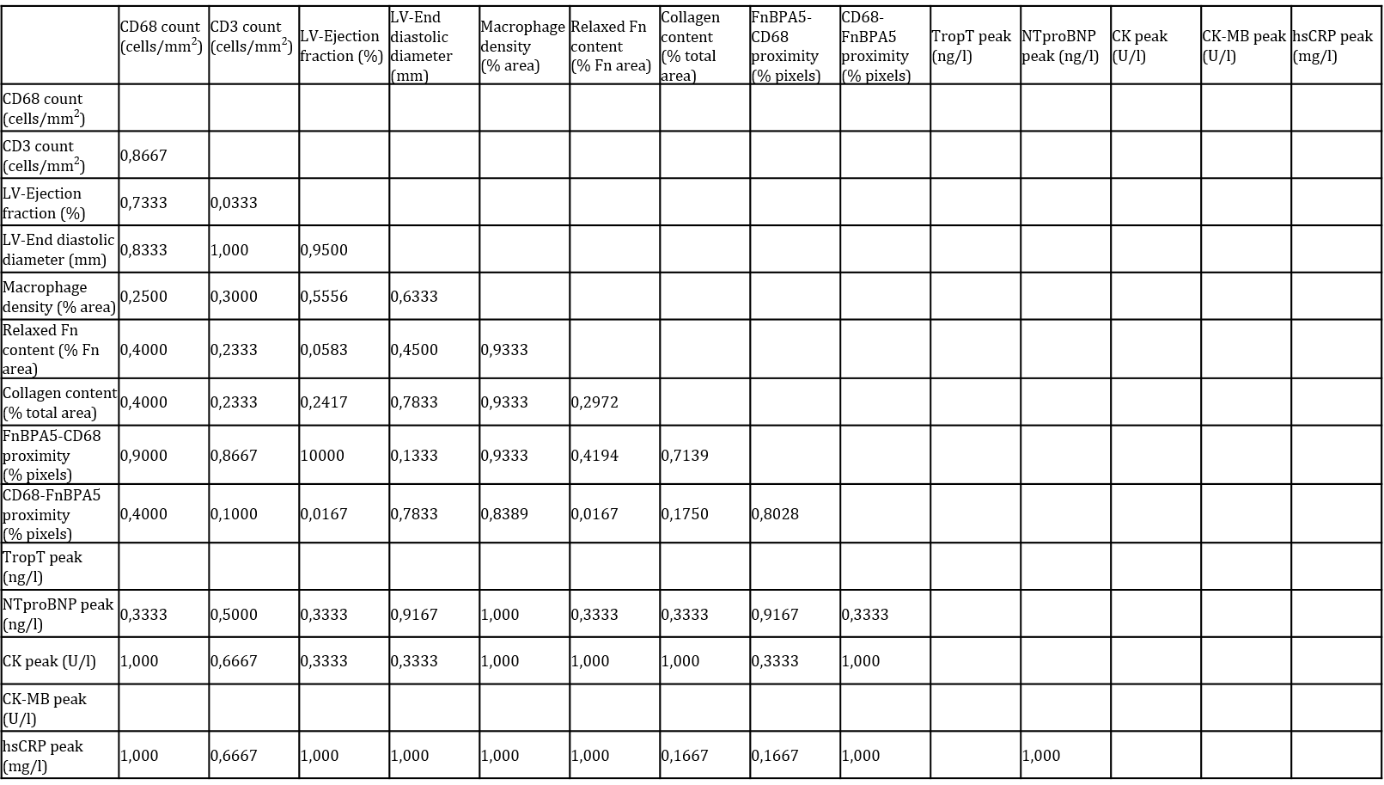
**

**DCMi (N=12):**

**
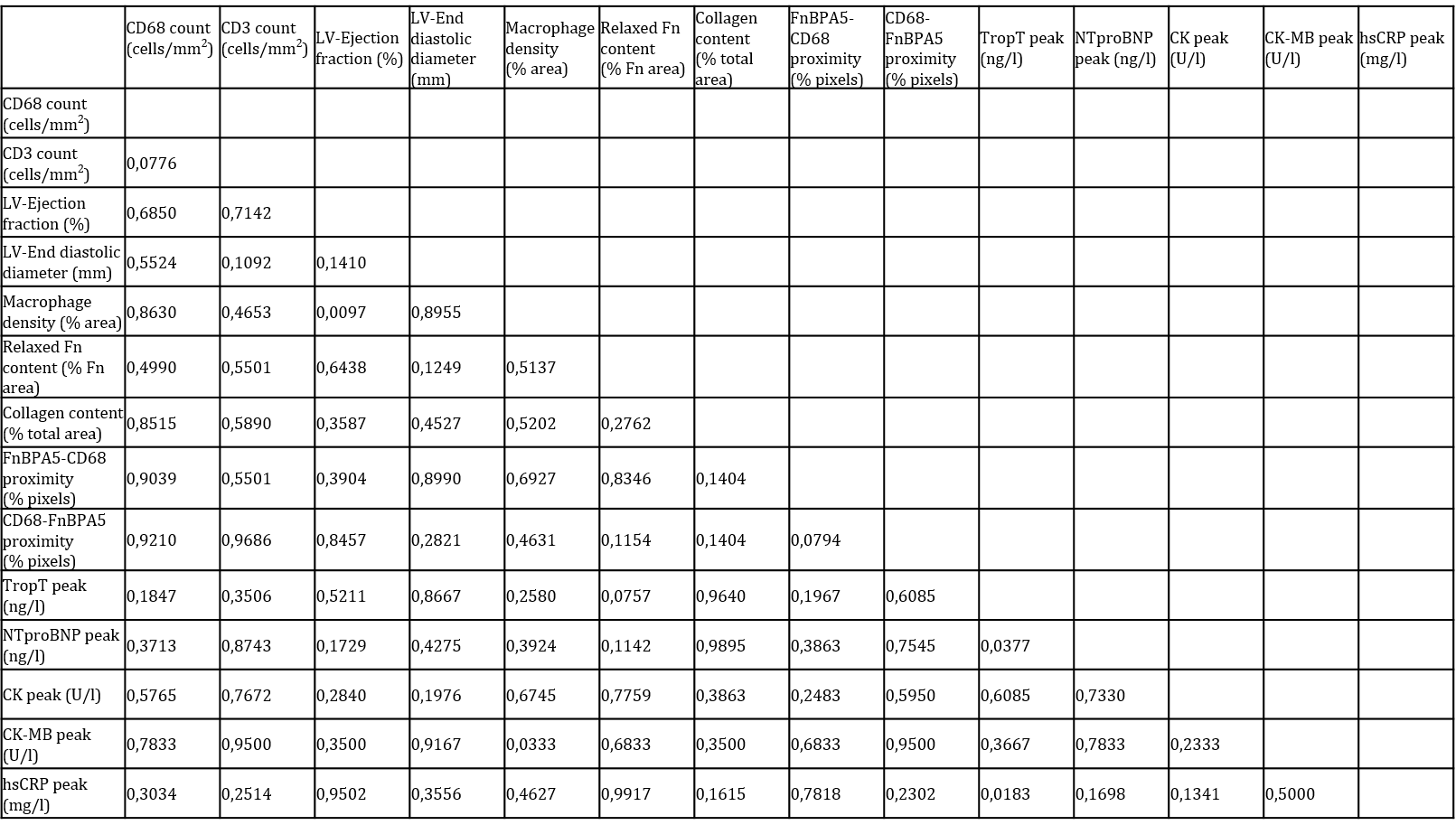
**

**COVID-19 (N=4):**

**
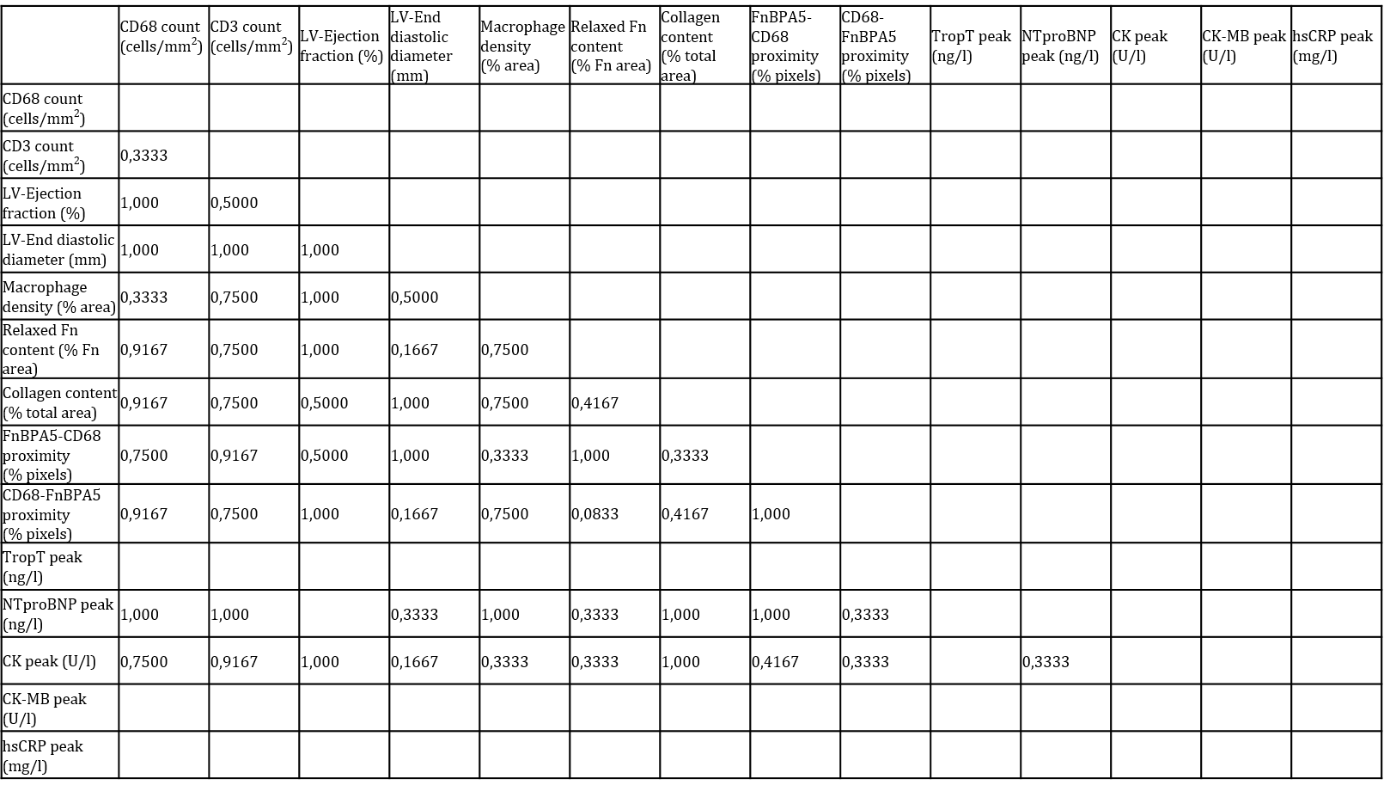
**

### Supplements to Materials and Methods: histological stainings:

Firstly, the tissue sections stored at -20 °C were air dried at room temperature (RT) for 20 min. The sections were then washed with PBS. To stain for the FnBPA5/Scrambled FnBPA5 peptide, the sections were blocked with 1% BSA in PBS for 30 min at RT. The sections were then treated with 5 µg of Cy5-FnBPA5/Cy5-scrambled FnBPA5 peptide (1:200 dilution of stock solution). The sections were incubated at RT with the peptides for 1 h following which they were washed with PBS. To stain for the antibodies, the sections were first blocked with 5% solution of goat and donkey serum in 1% PBS for 30 min. Following the blocking step, the sections were incubated with primary antibodies (for details refer **Supp. Table 4**) in antibody diluent solution overnight at 4 °C. Secondary antibodies Alexa488 goat anti-mouse and Alexa555 donkey anti-rabbit antibodies were added at a dilution 1:200 in 5% solution of goat and donkey serum in 1% BSA in PBS and incubated for 2h at RT in dark. To stain the nuclei of cells, the sections were incubated with 1:1000 dilution of DAPI in distilled water for 15 min. To mount the slides Fluoromount-G was added and a glass cover slip (#0, 24x32 mm) was carefully placed on to the tissue. The slides were dried overnight and stored in the dark for microscopic imaging subsequently.

**Table 4: List of markers and antibodies used:**

| **Antibody** | **Host, Reactivity** | **Dilution** | **Catalog #, Manufacturer** |
| --- | --- | --- | --- |
| CD68 | Mouse anti-Human CD68 | 1:100 | BSB5291, BioSB |
| CD3 | Mouse anti-Human CD3 | 1:50 | 21810031, Immunotools GmbH |
| Fibronectin | Rabbit anti-Human Fn | 1:200 | ab23750, Abcam |
| Alpha SMA | Mouse anti-Human ASMA | 1:100 | M0851, DAKO |
| CD31 | Rabbit anti-Human CD31 | 1:50 | PA5-16301, Invitrogen |
| Alpha actinin | Rabbit anti-Human alpha actinin | 1:100 | Ab68167, Abcam |
| MMP2 | Mouse anti-Human MMP2 | 1:100 | 351300Z, Invitrogen |
| MMP9 | Rabbit anti-Human MMP9 | 1:100 | Ab283575, Abcam |

**Table 5: Comparison of clinical parameters (shown in yellow) and experimental parameters (shown in green) in tissues from patients showing loci of macrophage crowding vs macrophage non-crowded myocardial tissues across patient groups (MC, DCM, DCMi and COVID-19)**

| **Šídák's multiple comparisons test – Patient tissues with macrophage-crowded loci versus non-crowded** | | | |
| --- | --- | --- | --- |
|  |  |  |  |
|  | **Mean**  **(crowded, N=5)** | **Mean**  **(non-crowded, N=30)** | **Significance (p≤0.05)** |
| **Clinical diagnosis** - **CD68 count (cells/mm^2^)** | **55.7** | **31** | ns |
| **Clinical diagnosis** - **CD3 count (cells/mm^2^)** | **31.3** | **14.5** | ns |
| **Clinical diagnosis - LV-Ejection fraction (%)** | 42.3 | 36.4 | ns |
| **Clinical diagnosis - LV-End diastolic diameter (mm)** | 57.8 | 57.8 | ns |
| **Macrophage density**  **(mean % of CD68+ pixels per square grid area)** | **0.8** | **0.09** | ns |
| **Relaxed to total fibronectin pixel content**  **(%)** | **7.2** | **3.6** | ns |
| **Collagen fiber content**  **(% of total area)** | 6.8 | 11.3 | ns |
| **TropT peak (ng/l)** | 92.5 | 232.1 | ns |
| **NTproBNP peak (ng/l)** | 4096 | 3341 | ns |
| **CK peak (U/l)** | 443.2 | 160.9 | ns |
| **CK-MB peak (U/l)** | 35.4 | 27.6 | ns |
| **hsCRP peak (mg/l)** | **167.1** | **16.4** | ns |
